# Field sampling of fig pollinator wasps across host species and host developmental phase: Implications for host recognition and specificity

**DOI:** 10.1002/ece3.10501

**Published:** 2023-09-11

**Authors:** Aafke Oldenbeuving, Adalberto Gómez‐Zúniga, Ximena Florez‐Buitrago, Ana M. Gutiérrez‐Zuluaga, Carlos A. Machado, Tom J. M. Van Dooren, Jacques van Alphen, Jacobus C. Biesmeijer, Edward Allen Herre

**Affiliations:** ^1^ Naturalis Biodiversity Center Leiden The Netherlands; ^2^ Institute of Environmental Sciences (CML) Leiden University Leiden The Netherlands; ^3^ Smithsonian Tropical Research Institute Panama Republic of Panama; ^4^ Department of Plant Sciences McGill University Ste‐Anne‐de‐Bellevue Quebec Canada; ^5^ Department of Biology Utah State University Logan Utah USA; ^6^ Department of Biology University of Maryland College Park Maryland USA; ^7^ CNRS, Institute of Ecology and Environmental Sciences Paris France

**Keywords:** diversification, Ficus, host‐recognition, pollinators, specificity

## Abstract

Previous genetic studies of pollinator wasps associated with a community of strangler figs (*Ficus* subgenus *Urostigma*, section *Americana*) in Central Panama suggest that the wasp species exhibit a range in host specificity across their host figs. To better understand factors that might contribute to this observed range of specificity, we used sticky traps to capture fig‐pollinating wasp individuals at 13 *Ficus* species, sampling at different phases of the reproductive cycle of the host figs (e.g., trees with receptive inflorescences, or vegetative trees, bearing only leaves). We also sampled at other tree species, using them as non‐*Ficus* controls. DNA barcoding allowed us to identify the wasps to species and therefore assign their presence and abundance to host fig species and the developmental phase of that individual tree. We found: (1) wasps were only very rarely captured at non‐*Ficus* trees; (2) nonetheless, pollinators were captured often at vegetative individuals of some host species; (3) overwhelmingly, wasp individuals were captured at receptive host fig trees representing the fig species from which they usually emerge. Our results indicate that wasp occurrence is not random either spatially or temporally within the forest and across these hosts, and that wasp specificity is generally high, both at receptive and vegetative host trees. Therefore, in addition to studies that show chemicals produced by receptive fig inflorescences attract pollinator wasps, we suggest that other cues (e.g., chemicals produced by the leaves) can also play a role in host recognition. We discuss our results in the context of recent findings on the role of host shifts in diversification processes in the *Ficus* genus.

## Graphical abstract

We sampled fig‐pollinating wasp individuals in a natural Neotropical *Ficus* community, at different phases of the reproductive cycle of the host figs (e.g., trees with receptive inflorescences, or vegetative trees, bearing only leaves), and compared with other tree species, using them as non‐*Ficus* controls. Our results indicate (1) that wasp occurrence is not random either spatially or temporally within the forest and across these hosts and (2) that wasp specificity is generally high, both at receptive and non‐receptive host trees. Therefore, in addition to studies that show chemicals produced by receptive fig inflorescences attract pollinator wasps, we suggest that other cues (e.g., chemicals produced by the leaves) can also play a role in host recognition.

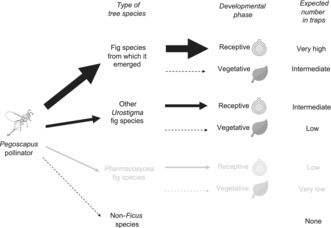

## 1 INTRODUCTION

Pollinators affect diversification in many plants (Grant, 1949; Kay & Sargent, 2009; Van Der Niet et al., 2014). In species with animal pollen vectors, genetic isolation of plant populations or species can be maintained, or broken down depending on whether the degree of pollinator specificity is high or low, respectively (Kiester et al., 1984; Moe & Weiblen, 2012; Moreira‐Hernández & Muchhala, 2019). With greater host specialization, pollinators limit the opportunities for hybridization between plant species (Ayasse et al., 2010; Byers et al., 2014; Wang et al., 2016; Whitehead & Peakall, 2014). Pollinator‐mediated speciation is therefore expected to be a relatively more common process in plant groups that are associated with more specialized pollinators (Moe et al., 2013; Schiestl, 2012).

One example of a functionally diverse and species‐rich plant genus with highly specific pollinators are fig trees (genus *Ficus*; around 850 species globally, Berg et al., 2005). The genus *Ficus* appears to have originated roughly 60–80 MYA and is defined by the enclosed inflorescence (syconium = “fig”). Fig trees rely completely on minute and highly specialized fig‐pollinating wasps (Agonidae) to correctly recognize an appropriate receptive host, enter a syconium, and pollinate the flowers within it. For their part, the fig wasps can only reproduce inside the reproductive structures of figs (Galil & Eisikowitch, 1968). Female wasps pollinate and lay eggs in female flowers that then form galls in which wasp offspring develop (Galil & Eisikowitch, 1968). Due to the synchronized development of syconia within flowering individuals in nearly all *Ficus* species, there are usually no receptive flowers available for fig wasps at eclosion in their natal tree (Janzen, 1979). Given this synchronized development within a host tree and the short lifespan of adult pollinator wasps (around 2–3 days; Kjellberg et al., 1988; van Kolfschoten et al., 2022), most wasps need to travel great distances to encounter a receptive host and finding a suitable receptive host is challenging, especially when the density of host trees is low, as in monoecious neotropical figs (McKey, 1989; Todzia, 1986).

Large dispersal distances have been reported for fig pollinators (Ahmed et al., 2009; Nason et al., 1998), but how precisely they can encounter appropriate hosts (e.g., a receptive individual of the fig species from which she emerged) across what can be very large distances remains unknown. One of the cues that allows a fig pollinator to recognize an appropriate fig host has been identified as volatile chemicals emitted by receptive syconia (Bronstein, 1987; van Noort et al., 1989; Ware & Compton, 1994a). These chemicals appear to provide reliable information about both species identity of the fig as well as the reproductive phase (Cornille et al., 2012; Grison‐Pigé et al., 2002; Proffit & Johnson, 2009; Ware et al., 1993).

At a single location, in most cases, a single fig species seems to be pollinated by one or two wasp species, and each pollinator species is usually associated with only one fig species. However, an increasing number of examples of two fig species sharing a pollinator species are reported, allopatrically as well as sympatrically (Moe et al., 2011; Molbo et al., 2003; Yang et al., 2015). Further, population genetic studies have revealed that natural hybridization between *Ficus* species is not uncommon (Moe et al., 2011; Parrish et al., 2003; Satler et al., 2022; Wang et al., 2016; Wei et al., 2014).

From the tight and specific relationships generally observed between figs and their pollinators, scholars have inferred an evolutionary history of co‐diversification (Cruaud, Ronsted, et al., 2012; Ramirez, 1974; Wiebes, 1979). Indeed, on a macroevolutionary level studies show a co‐divergence between figs and pollinating wasps (Cruaud, Ronsted, et al., 2012; Herre et al., 1996; Silvieus et al., 2008). However, increased sampling, collectioning of molecular data from multiple loci or genomic data, and improved co‐phylogenetic analyses, have eroded the support for this binary scenario of strict co‐adaptation and co‐speciation (Cook & Segar, 2010; Cruaud, Cook, et al., 2012; Hembry & Althoff, 2016; Herre et al., 2008; Satler et al., 2019, 2020, 2022). These more recent analyses indicate that pollinator and fig phylogenies are often incongruent at lower taxonomic levels (species within *Ficus* sections or within wasp genera), and there is increasing evidence for regular hybridization between figs (Gardner et al., 2023; Jackson et al., 2008; Machado et al., 2005; Satler et al., 2019, 2020, 2022; Wang, Zhang, et al., 2021; Wilde et al., 2020). And a recent co‐phylogenetic analysis of a well‐studied community of Neotropical fig species and their associated pollinator species suggested that host‐shift events have been as common as strict co‐speciation events (Satler et al., 2019).

A key general question is what mechanism underlies different degrees of pollinator specificity, both within and across fig species. Evolutionary and ecological patterns found in the fig‐wasp mutualism suggest that this mechanism balances high specificity of and occasional errors by pollinators. An important part of the answer can be found in determining how volatile chemical signals can play an ecological role in guiding pollinators both temporally and spatially to their appropriate fig host. Studies on host recognition by fig wasps therefore need to be refined and focused to documenting patterns, especially in diverse, naturally occurring fig communities and guided by testable hypotheses. Here we document presence, relative abundances, and species identities of pollinator individuals collected in a natural community of strangler figs (subgenus *Urostigma*, section *Americana*; pollinated by fig wasps from the genus *Pegoscapus*) in Neotropical forest in the vicinity of the Panama Canal. Our setup allows for testing the following two hypotheses.

Host‐searching pollinator individuals will be more abundant at (and presumably more attracted to) receptive fig trees belonging to the *Ficus* species from which it emerged than they are to: receptive trees belonging to other *Ficus* species, or vegetative trees of any *Ficus* species, or non‐*Ficus* trees.

In addition to volatiles produced by the receptive syconia, other signals (e.g., volatiles produced by leaves or other plant parts) also promote pollinator wasp attraction.

**FIGURE 1:**
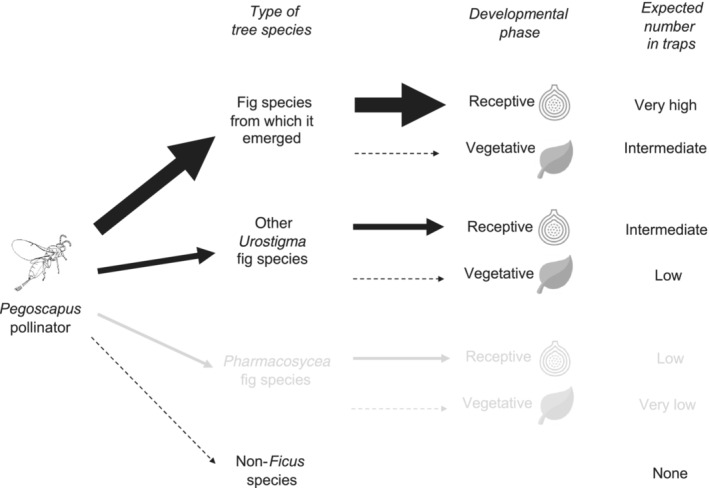
Expected numbers of trapped *Pegoscapus* pollinator individuals at different *Ficus* species and host developmental phases based on our hypotheses. Widths of the arrows represent the hypothesized relative attractiveness of each type of tree. Arrows with dashed lines represent pollinator choices resulting in pollinator fitness zero. If pollinators are most abundant at receptive trees belonging to the fig species from which they emerged (H1) and are attracted to other volatiles than those from receptive syconia (H2), we expect that the number of trapped pollinators increases from non‐*Ficus* trees, to vegetative *Ficus*, to receptive *Ficus* trees as well as from other *Urostigma* species to the *Urostigma* species from which it emerged. Parts in gray represent predictions that we do not directly address in this paper, based on the assumption that phylogenetic distance predicts similarity in fig volatile bouquets and thus to which pollinator species is more likely to be attracted.

Combining these hypotheses, we predict that the number of *Pegoscapu*s pollinator individuals that can be trapped within a forest containing a diverse community of fig trees increases dramatically from non‐*Ficus* trees to vegetative *Urostigma* trees, with the greatest number trapped on receptive *Urostigma* trees. We also predict, for any *Pegoscapus* species, the number of trapped individuals will routinely be highest on the fig species from which the pollinator emerged compared to other *Urostigma* species, regardless of the host developmental phase. We also predict that pollinators occasionally make mistakes, and then are found either on vegetative individuals belonging to the *Ficus* species from which they emerged or on receptive individuals belonging to closely related *Ficus* species. Our predictions are summarized in Figure 1.

## 2 MATERIALS AND METHODS

### 2.1 Fig species and research area

Pollinator trapping for this study was carried out at trees on the shores of lake Gatun in the Barro Colorado Nature Monument in Central Panama. These shores are covered in moist seasonal forest. Fig species occurring in this area belong to two subgenera of *Ficus*. Trees from the subgenus *Urostigma* (section *Americana*) are known as strangler figs and they are pollinated by wasps of the genus *Pegoscapus*. Trees from the subgenus *Pharmacosycea* (section *Pharmacosycea*) are known as free‐standing figs and are pollinated by wasps of the genus *Tetrapus*. Pollinators were trapped at 13 *Urostigma* fig species (varying from 1 to 5 individual trees per fig species, see Table 2). From published and ongoing studies, the *Pegoscapus* species that commonly and successfully develop in and emerge from these fig species are well characterized. In most cases one pollinator species is strictly associated with a single fig species. However, *Pegoscapus gemellus* A has been consistently reared from two species of figure (*F. bullenei* and *F. popenoei*), and two species of pollinator have been consistently reared from *F. obtusifolia* (*P. hoffmeyeri* A and *P. hoffmeyeri* B) (Machado et al., 2005; Molbo et al., 2003). More recently, *P. insularis* has been reared from syconia from both *F. colubrinae* and *F. perforata* (Satler et al., 2019), whereas it used to be reared only from *F. perforata* (Machado et al., 2005; Molbo et al., 2003). Either it was missed in older surveys, or this pollinator species has expanded to an additional host fig species. Furthermore, in recent years the pollinator of *F. paraensis* (*P. herrei*) seems to have been replaced by an unknown pollinator species (unpublished data from Herre, Machado, and Piatscheck). The current fig‐pollinator associations are listed in Table 1.

**TABLE 1:** Overview of the *Urostigma* fig species at which pollinators were trapped. For all fig species, the *Pegoscapus* pollinator species that develop in and emerge from them are known. A section sign (§) indicates *Pegoscapus* species known to develop in two host species. A minus sign (−) indicates a *Pegoscapus* species that have become rare over past 20 years. A plus sign (+) indicates a *Pegoscapus* species that have become more common in this *Urostigma* species over the past 20 years.

*Urostigma Ficus* species	*Pegoscapus* pollinator species
*F. bullenei*	*P. gemellus* A^§^, *P. gemellus* C
*F. citrifolia*	*P. tonduzi*
*F. colubrinae*	*P. orozcoi* ^−^, *P. insularis* ^§+^
*F. costaricana*	*P. estherae*
*F. dugandii*	*P. longiceps*
*F. near trigonata*	*P. lopesi*
*F. nymphaefolia*	*P. piceipes*
*F. obtusifolia*	*P. hoffmeyeri* A, *P. hoffmeyeri* B
*F. paraensis*	*P. herrei* ^−^, *P. ‘ex paraensis’* ^+^
*F. perforata*	*P. insularis* ^§^
*F. pertusa*	*P. silvestrii*
*F. popenoei*	*P. gemellus* A^§^, *P. gemellus* B
*F. trigonata*	*P. grandii*

### 2.2 Pollinator trapping on *Urostigma* fig hosts and non‐*Ficus* trees

Pollinator individuals were trapped in both receptive and vegetative *Urostigma* fig trees, as well as in non‐*Ficus* controls (the latter during one field season). We used sticky traps made of yellow plastic sheets (20 × 10 cm) which were covered with odorless non‐drying glue (TangleTrap) on both sides. For each trapping event, four sticky traps facing haphazard directions were placed at a tree at the lake side with accessible branches reaching down. Within the tree they were placed as far apart as possible and as close to the leaves as possible (in these fig species leaves and syconia grow next to each other) and always between a height of 1 or 2 m above lake‐level. For each trapping event, traps remained for at least 24 h up to a maximum of 6 days. After exposure, the traps were taken to the lab where the *Pegoscapus* individuals were counted using a stereoscope. The *Pegoscapus* individuals could not be identified as pollinator species by eye but could be distinguished from *Tetrapus* individuals. The total number of *Pegoscapus* individuals collected per host tree per 24 h was calculated for each trapping event.

A fig tree was defined to be in the vegetative phase when it had no observable syconia but only green leaves. Fig trees bearing syconia are not always in the receptive phase, though. This is only when the female flowers are receptive, and pollination is possible. The ostiole, a layered entrance to the flowers, loosens and permits access during this developmental phase (Galil & Eisikowitch, 1968). It usually takes a few days or week for all receptive syconia to be pollinated, but this can take longer if pollinator availability is low (Anstett et al., 1996; Khadari et al., 1995). Whether a host tree was receptive at the moment of sampling was determined with hindsight and was done as follows. Each day, 10 syconia were collected haphazardly and checked for the presence of living pollinators or dead pollinators inside. When living fig pollinators were observed inside one or more syconia or when the number of syconia with dead pollinators inside had increased compared to the previous day, the host tree was determined to be receptive. This is probably a conservative definition of receptivity, there may be more days during with a host tree is receptive. However, any other way of determining receptivity, for example, by observing the ostioles, was considered less objective.

For the non‐*Ficus* control group, we haphazardly selected 15 trees. We did not have the expertise to identify these non‐*Ficus* trees to species, but they all belonged to different tree species. We further made sure that these trees bore only leaves, and no flowers or fruits during the trapping events. And we further assured that the distribution of the non‐*Ficus* trees across the nature reserve was similar to that of the fig trees in this study. To reduce the probability of trapping *Pegoscapus* pollinators going to a nearby fig host at our control trees, we only chose non‐*Ficus* trees for which the closest observable fig host was at least 30 m away. This distance was chosen based on a small pilot study in which we found no pollinators at a distance of 20 m from a fig host. A summary of the trapping events is found in Table 2.

**TABLE 2:** Overview of the number of trapping events (event = 4 sticky traps up for an “*x*” number of days), and the number of barcoded pollinator individuals per fig species per developmental phase. Note that the number of identified pollinators from traps is not representative of the total number of trapped pollinators which are presented in the results.

Fig species	Sticky trap sampling				DNA‐barcode sampling			
	Number of trees		Number of trapping events		Number of trees		Number of identified pollinators	
	Receptive	Vegetative	Receptive	Vegetative	Receptive	Vegetative	Receptive	Vegetative
*Ficus bullenei*	3	7	10	231	2	3	35	55
*F. citrifolia*	3	5	14	29	2	1	45	27
*F. colubrinae*	2	3	8	87	1	0	16	0
*F. costaricana*	1	1	3	3	0	0	0	0
*F. dugandii*	1	1	10	5	1	0	28	0
*F. near trigonata*	3	1	9	2	2	1	15	9
*F. nymphaefolia*	3	3	12	46	0	0	0	0
*F. obtusifolia*	5	5	11	25	2	3	40	76
*F. paraensis*	1	1	4	5	1	0	26	0
*F. perforata*	1	2	12	49	1	1	32	3
*F. pertusa*	1	1	8	4	1	0	10	0
*F. popenoei*	3	7	22	76	3	3	51	45
*F. trigonata*	1	2	3	40	2	0	18	0
Total	28	39	126	602	18	12	316	215
Non‐*Ficus* trees	15		60		0		0	

### 2.3 DNA barcoding

For comparisons at pollinator species level, a subset of the trapped fig pollinators was collected for DNAbarcoding. Where possible we tried to collect up to 20 individuals per host for both the receptive and vegetative phase. The pollinators were cleaned of glue by rinsing them in synthetic turpentine for about 15 min and next in water with soap for 1 min. After cleaning, pollinators were stored in 90% ethanol until further processing. Fig wasp DNA from single individuals was extracted using the Gentra PureGene Tissue Kit (Qiagen) with minor modification.

A total of 503 base pairs of CO1 were PCR amplified using the primers NewCOI_DEG_668_F (CTC TGG RGG KGG TGA TCC AA) and NewCOI_DEG_1171_R (AAA ATW GCA TAN ACW GCN CCT A). These degenerate primers were designed using assembled transcriptomes from two species of pollinator (*Pegoscapus* sp. ex. *F. dugandii*, and *F. petiolaris*; C. A. Machado, unpublished). Transcriptomes were assembled using Trinity (Grabherr et al., 2011) and previously published COI fig wasp sequences (Machado et al., 2001, 2005; Molbo et al., 2003) were blasted to the assembly to identify mtDNA scaffolds that included COI. Those scaffolds were then used to generate a battery of primer pairs for COI that were tested to identify pairs that worked consistently across multiple species. Primers NewCOI_DEG_668_F and NewCOI_DEG_1171_R were the best pair that generated consistent clean PCR bands across all species tested. PCR reactions were performed in 20 μL containing Buffer 1×, 0.25 mM of each dNTP, 1 mM of MgCl_2_, 0.25 μM of each primer, 1 U of Taq polymerase QIAGEN, and 1 μL of genomic DNA. Amplifications were carried out in a thermal cycler programmed as follows: 3 min at 95°C for 1 cycle; 30 s at 95°C, 45 s at 57°C (decreasing 1°C per cycle), and 1 min at 72°C for 15 cycles (Touchdown PCR); 30 s at 95°C, 45 s at 47°C, and 1 min at 72°C for 20 cycles; 5 min at 72°C for one terminal cycle. 5 μL of each PCR reaction mixture were electrophoresed in a 1% agarose gel. Gels were stained with GelRed® and bands visualized under ultraviolet illumination. Amplified products were purified and sequenced in both directions at Macrogen (Korea).

Barcoding was conducted using phylogenetic analyses (Figures S1–S11). The pollinator CO1 sequences were aligned using a reference data matrix composed of all *Pegoscapus* sp. and *Tetrapus* sp. COI sequences found in the NCBI GenBank database (Benson et al., 2005). Aligned sequences from each pollinator individual were first translated using the invertebrate mitochondrial genetic code to confirm they were coding sequences and not nuclear pseudogenes. None of the sequences showed evidence of pseudogenization or frame shifts. Aligned fig pollinator data from each fig species was then analyzed in Geneious Prime v2021.2.2 using the neighbor‐joining algorithm with Tamura‐Nei distances (Tamura & Nei, 1993). DNA sequences were assigned the species name associated with GenBank reference sequences they clustered with within the phylogeny, typically corresponding to sequences from the GenBank reference with <2% divergence. In cases where sequences did not cluster with any GenBank reference sequence, they were named “new sp.” and their closest reference sequence or clade was noted. COI sequences were deposited in GenBank (accession numbers OR288903—OR289513). The total number of identified individuals per fig species is listed in Table 2.

### 2.4 Statistical analyses

R was used for all statistical analyses and estimates obtained (R version 4.3.1). First, the number of trapped *Pegoscapus* pollinators on different non‐*Ficus* trees, vegetative *Urostigma* fig trees, and receptive *Urostigma* fig trees were compared using zero‐inflated Poisson mixed models (glmmTMB package; Brooks et al., 2017). These models assume that observations are draws of mixtures of additional zero counts and counts following Poisson distributions (of which a fraction of observed counts will be zero as well). The mixing proportions are determined by probabilities which are modeled with logistic regressions. These regressions are called the zero‐inflation model. The Poisson model is called the conditional model. The most elaborate mixed model fitted to our data contained a zero‐inflation model with a fixed effect of tree type and random fig species effects. The conditional model for mean counts contained an offset for the number of days a trap was put (the offset was log; number of days), a fixed categorical effect of host type and random species and date effects. This model and simplified models with fixed and random effects removed were compared using AIC (Claeskens & Hjort, 2008). We report tail probabilities of hypothesis tests on the significance of tree type fixed effects in the model with the lowest AIC. Likelihood ratio tests where we simulated the null hypothesis distribution using parametric bootstrap were impossible because simplified null models required did not fit the data. We therefore report *z*‐tests on the difference parameters of the tree type effect in the model with lowest AIC. Using the model with the lowest AIC we computed 95% confidence intervals for model parameters and of differences between predicted counts on different host types using Tukey‐corrected confidence intervals for pairwise differences.

Second, we also wanted to inspect the difference between non‐*Ficus*, vegetative, and receptive trees at the level of fig species, and we did this for the four *Ficus* species at which we found pollinators during the receptive and vegetative phases and with at least one observation for each reproductive phase during which we counted more than a single pollinator (*Ficus bullenei*, *F. citrifolia*, *F. obtusifolia*, and *F. popenoei*). Here we used zero‐inflated Poisson mixed models as well (with random date effects, glmmTMB package; Brooks et al., 2017). However, these did not reach convergence. We therefore used zero‐inflated Poisson generalized linear models using the zeroinfl() function of library pscl (Jackman, 2020; Zeileis et al., 2008). Models with joint fixed tree type and date effects were usually singular or failed to fit. At the level of *Ficus* species, the number of days on which receptive and vegetative trees were sampled was limited, such that effects were not well crossed. Models with fixed date effect would contain large numbers of parameters tending to overfit the data and had very low precision of individual parameter estimates due to separation. Therefore, in the models compared, date effects were removed. The zero‐inflated model which had lowest AIC for each species contained tree type effects in the logistic regression for zero‐inflation and the conditional Poisson model. For each fig species, this model was used to calculate 95% confidence intervals for the difference between tree types for each fig species.

Third, we used the barcoding results to compare the specificity of pollinators on different vegetative and receptive fig trees using binomial generalized linear mixed models (lme4 package; Bates et al., 2014) for the probability that a pollinator was found on its preferred host, with random pollinator species effects and a fixed effect interaction of tree type and host species. Models with random effects did not converge and therefore binomial generalized linear models were used with the same fixed effects and fixed effects of pollinator species. We compared this model with simplifications of it using AIC. Inspection of the parameter estimates revealed that pollinator species effects had to be removed because they overfitted the data (separation and no precision of parameter estimates). Among the models with tree type and host species fixed effects, the one with lowest AIC was used to report likelihood ratio tests and calculate 95% confidence intervals for the differences between receptive and vegetative trees.

Finally, we wanted to obtain predictions of counts at pollinator species level, which were not directly measured. For this the barcoding results, and the counts of trapped *Pegoscapus* pollinators were jointly used. Bootstrap resampling from both datasets was used to generate 200 count datasets, and 200 relative abundance datasets. Multiplying these datasets yielded 200 datasets of the number of pollinators per *Ficus* species for receptive usual hosts, receptive other hosts, vegetative usual hosts, and vegetative other hosts. The 95% confidence intervals based on these resampled datasets were used to compare numbers of pollinators. These intervals were made for six species that are known as the associated pollinators of the fig species we analyzed at the level of fig species.

## 3 RESULTS

### 3.1 Pollinator presence at receptive and vegetative *Ficus* host trees, or at non‐*Ficus* controls

**FIGURE 2:**
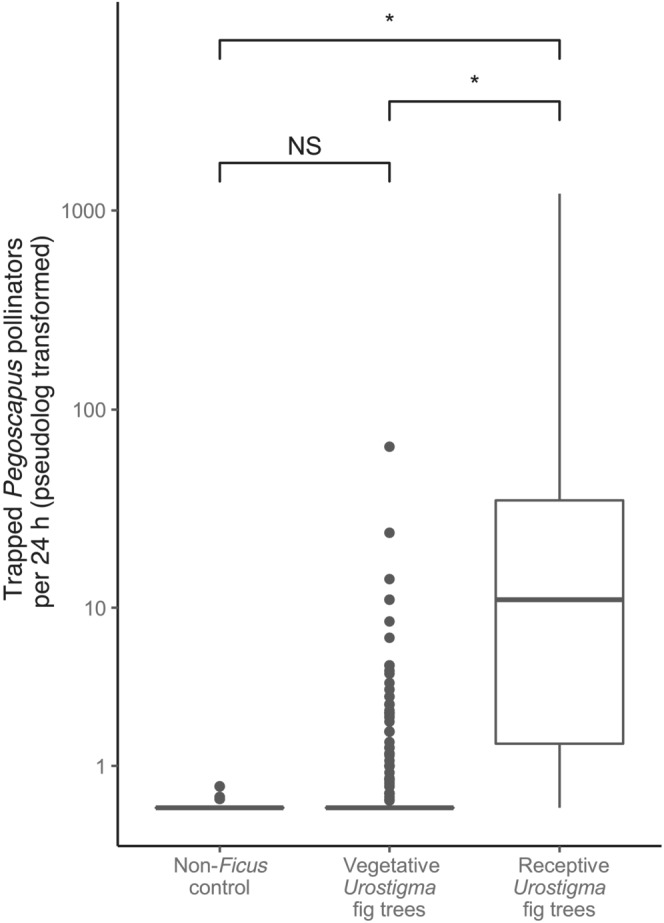
*Pegoscapus* pollinator individuals trapped across three types of trees. Far more *Pegoscapus* individuals are trapped at receptive *Urostigma* trees. Significant differences based on 95% confidence intervals of the model are indicated with “*,” and non‐significant differences with “NS.” Note the pseudolog transformation; a transformation mapping numbers to a signed logarithmic scale with a smooth transition to linear scale around 0.

*Pegoscapus* pollinators were abundantly trapped at receptive *Urostigma* trees; we carried out 126 trapping events lasting 150 days in total during which 7580 pollinators were trapped (Figure 2). *Pegoscapus* pollinators were rarely trapped on non‐*Ficus* trees; during the 60 trapping events lasting 287 days in total only four pollinators were caught (Figure 2). On vegetative *Urostigma* fig trees we trapped an intermediate number of *Pegoscapus* pollinators; during 602 trapping events lasting 1198 days 580 *Pegsocapus* pollinators were trapped (Figure 2). The model with the lowest AIC (i.e., with largest efficiency, best capacity to predict) combined conditional and zero‐inflated modeling. We found a significant fixed effect difference between tree receptive and non‐*Ficus trees* (*z* = 2.96, *p* = .003), and random effects of fig species and trapping date in the conditional model and a fixed tree type effect in the logistic regression for the zero inflation (difference receptive vs. non‐*Ficus z* = −4.20, *p* < .001). The 95% confidence intervals for receptive *Urostigma* trees were 1.00 to 2.48 for the conditional model parameters, and −5.46 to −2.47 for logistic regression parameters, making the number of trapped pollinators at receptive *Urostigma* trees to be significantly higher compared to both non‐*Ficus* trees, and vegetative *Urostigma* trees. The statistical analyses show no difference in the number of trapped pollinators at non‐*Ficus* trees and vegetative hosts (95% c.i. for conditional model parameters non‐*Ficus* = −6.00 to 0.09, vegetative *Urostigma* trees = −2.08 to −0.454, and for logistic regression parameters: non‐*Ficus* = −0.52 to 3.73, vegetative *Urostigma* trees = 0.42 to 1.05).

**FIGURE 3:**
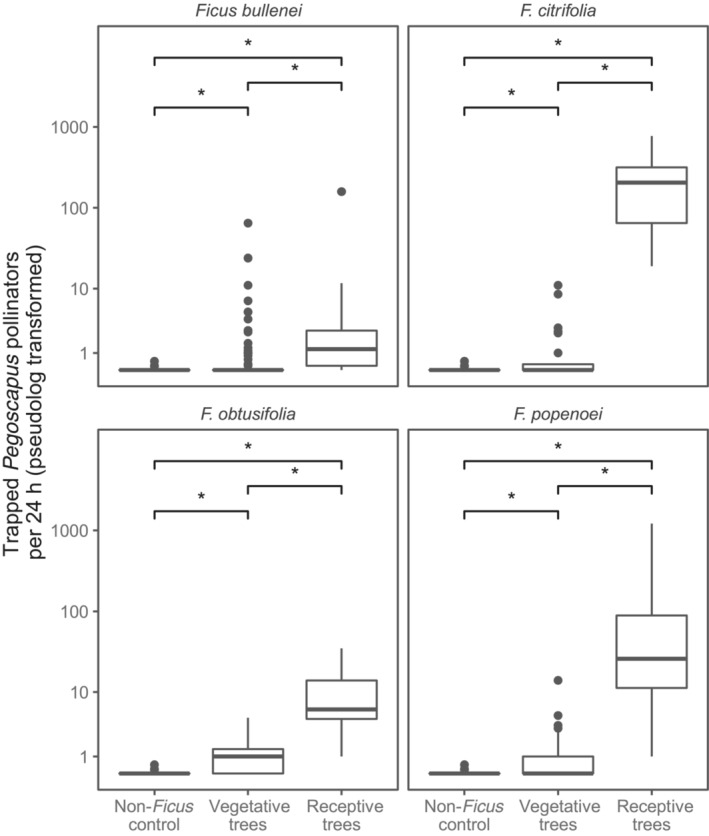
*Pegoscapus* pollinators trapped at four target *Urostigma* fig species. The number of trapped individuals at the non‐*Ficus* trees also plotted in each panel for comparison. More pollinators are trapped at vegetative *Ficus* trees compared to non‐*Ficus* controls. Significant differences, based on 95% confidence intervals calculated from the model for the number of trapped pollinators (see upper half of Table 2), are indicated with “*.” Note the pseudolog transformation; a transformation mapping numbers to a signed logarithmic scale with a smooth transition to linear scale around 0.

Nearly all *Pegoscapus* pollinators that were trapped at vegetative *Urostima* trees were caught in traps on one of the following four fig species: *Ficus bullenei*, *F. citrifolia*, *F. obtusifolia*, and *F. popenoei*. We therefore specifically inspected these species for differences between receptive, vegetative, and non‐*Ficus* trees (Figure 3). The 95% confidence intervals of the model parameters are summarized in Table 3. In each fig species, we found, as predicted, that more *Pegoscapus* pollinators were trapped at receptive trees compared to both non‐*Ficus*, and vegetative trees. Besides, in these four fig species the number of trapped pollinator individuals in vegetative trees is higher than the number of trapped pollinator individuals at non‐*Ficus* trees as well.

**TABLE 3:** 95% confidence intervals for receptive, vegetaive, and non‐*Ficus* trees compared within the four target fig species. Intervals are calculated from a zero‐inflated count model with random effects on both the mean count *λ* of the Poisson and the probability *p* of the binomial. Confidence intervals matching our predictions are indicated with "*", and confidence intervals not matching with "NS".

	95% interval for	Predicted interval	*Ficus bullenei*	*F. citrifolia*	*F. obstusifolia*	*F. popenoei*
Modeled difference in number of trapped pollinators	Receptive trees minus non‐*Ficus* trees	>0	2.67 to 6.79*	5.30 to 9.41*	2.40 to 6.53*	4.35 to 8.44*
	Vegetative trees minus non‐*Ficus* trees	>0	0.91 to 5.03*	0.34 to 4.48*	0.33 to 4.47*	0.23 to 4.34*
	Receptive trees minus vegetative	>0	1.54 to 1.99*	4.67 to 5.22*	1.79 to 2.35*	3.94 to 4.28*
Modeled difference in probability of trapping zero pollinators	Receptive trees minus non‐*Ficus* trees	<0	−5.73 to −0.41*	−924,454 to 92002^NS^	−10,430 to 10,380^NS^	−7379 to 7336^NS^
	Vegetative trees minus non‐*Ficus* trees	<0	−2.29 to 2.08^NS^	−4.24 to 0.18^NS^	−5.47 to −0.090^NS^	−4.42 to −0.15*
	Receptive trees minus vegetative	<0	−4.64 to −1.28*	−9243 to 93044^NS^	−10,420 to 10,390^NS^	−7377 to 7339^NS^

### 3.2 DNA barcoding, species identification, and species specificity

**FIGURE 4:**
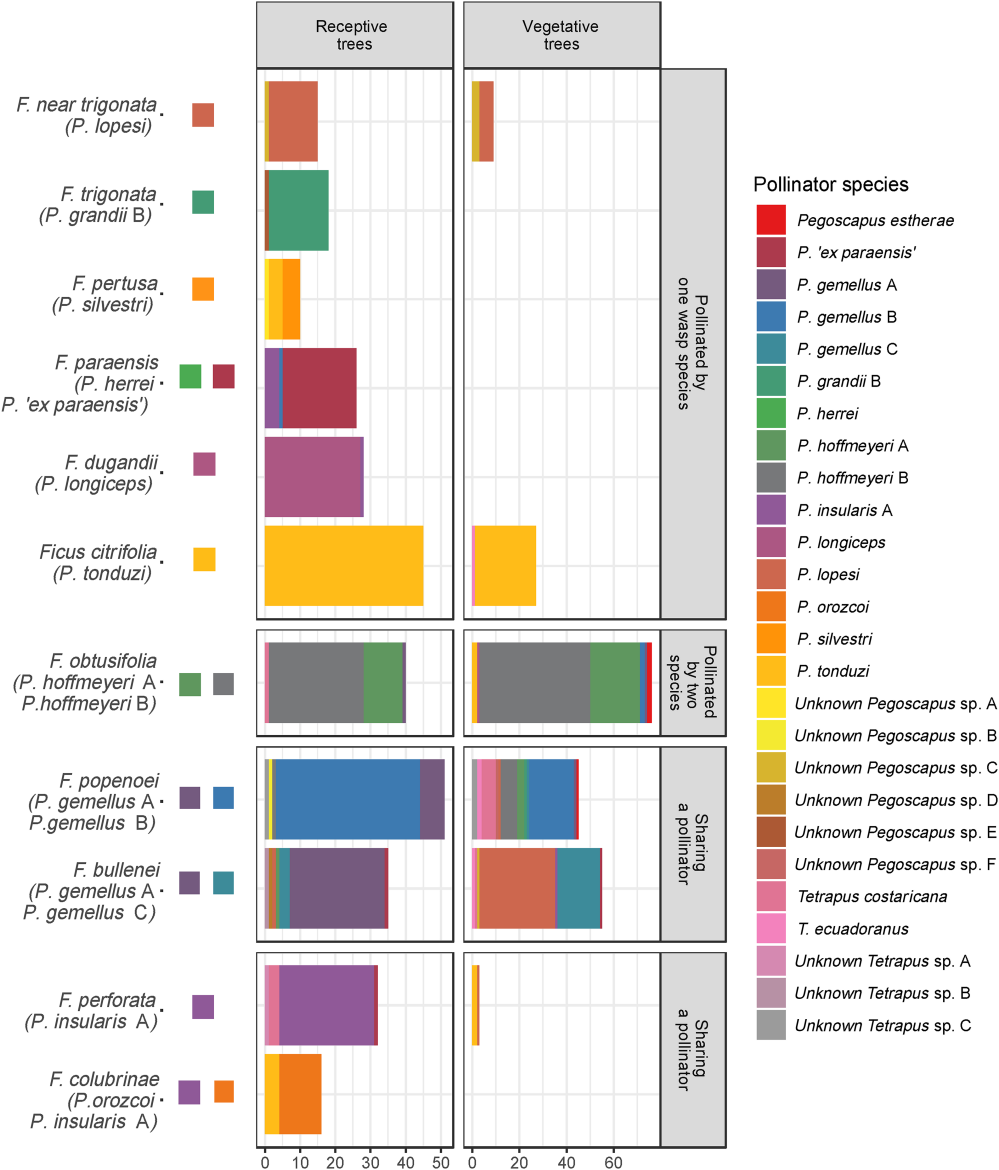
Species identifications of fig pollinators trapped at receptive and vegetative tree individuals belonging to 11 *Urostigma* fig species. The colored squares on the left side indicate the pollinator species usually emerging from these fig species. The majority of the pollinators identified at vegetative fig trees (right side) were trapped at their usual host species, and pollinators at receptive fig trees (left side) show an even higher specificity. The upper section represents six *Urostigma* fig species that have one associated *Pegoscapus* pollinator. The lower sections represent fig species that diverge from the 1‐to‐1 pattern. Note that, due to sampling bias, bar lengths are not representative of the number of trapped *Pegoscapus* individuals at the host trees.

In total 531 pollinator individuals trapped at 11 *Urostigma* fig species were barcoded and identified to species (Table 2, and Figure 4, Figures S1–S11). The barcoded pollinator individuals belong to 25 genetically distinguishable species, 16 of which were found in previously published and unpublished barcoding studies (Machado et al., 2001, 2005; Molbo et al., 2003). For these pollinator individuals (covering >98% of the barcoded individuals) the *Urostigma* species from which they usually emerge is known (Table 2).

284 of 316 of the pollinators caught at receptive *Urostigma* fig trees belong to the pollinator species that is commonly reared from such host (see Figure 4). Furthermore, a large majority (138 of 215) of pollinator individuals trapped on vegetative *Urostigma* trees were found at the fig species they usually emerge from (Figure 4). Binomial GLM were fitted to these data and found significant additive effects of tree type (*χ* ^2^(1) = 66.98, *p* < .001) and host species (*χ* ^2^(10) = 102.84, *p* < .001), and 95% confidence intervals were calculated. The results show that pollinator individuals trapped at receptive *Urostigma* trees belong more often to the pollinator species that emerged from it compared to individuals trapped on vegetative *Urostigma* trees (c.i. receptive = 1.14 to 2.59 and c.i. vegetative = −1.27 to −0.22). In each fig species except *Ficus citrifolia*, *Pegoscapus* pollinators were also incidentally trapped on receptive trees belonging to a fig species from which it not usually emerges. Also, seven *Tetrapus* pollinators, who are associated with fig species from a different fig section (*Pharmacosycea*), were caught on *Urostigma* trees (~2% of the barcoded individuals).

One remarkable find was that 32 of the 52 pollinator individuals identified from traps at vegetative *F. bullenei* trees belong to *P. lopesi* (Figure 3) a species that is known to pollinate *F. near trigonata*. Nearly all other barcoded individuals from *P. lopesi* had been trapped at *F. near trigonata*, and the pollinator seems to be very rare on other fig species, for example, one individual on *F. perforata* and one on *F. popenoei*. These 32 *P. lopesi* individuals were caught on two consecutive days at the same individual tree, and therefore we think we should be careful when interpreting these observations. We might have overlooked a nearby *F. near trigonata* tree releasing pollinators during our observation at *F. bullenei*. We included these wasps in the analyses because overall results were not significantly different when they were left out.

### 3.3 Pollinator abundances at species level

**FIGURE 5:**
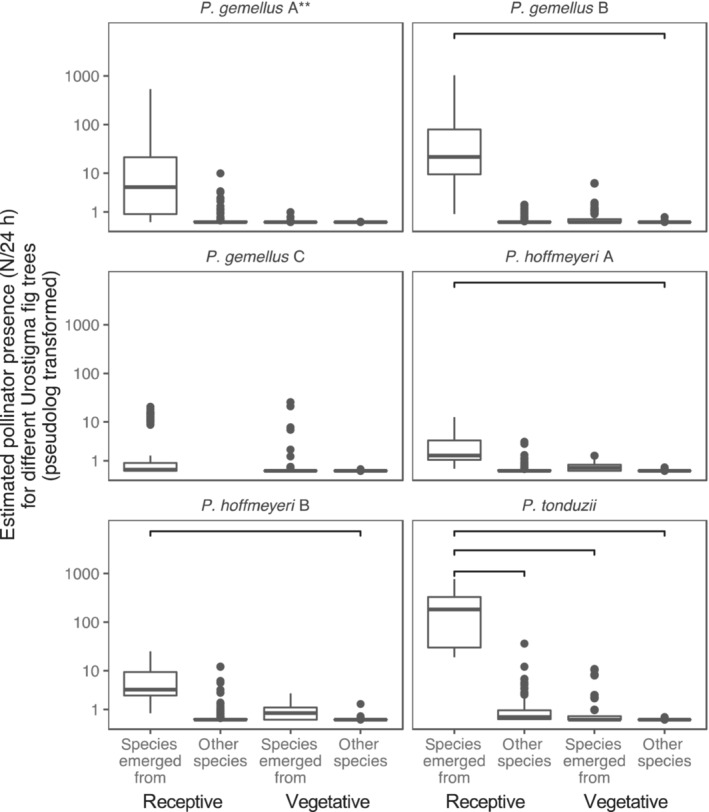
Estimates of the presence of six *Pegoscapus* pollinator species at four types of *Urostigma* fig trees. Pollinator estimates are highest for receptive fig trees belonging to the *Urostigma* species from which they regularly emerge. Besides, there seems to be a trend of higher pollinator estimates for other receptive fig trees as well as for vegetative fig trees that belong to their usual host species compared to other vegetative trees from other species. Estimates are based on bootstraps of the results presented in Figures 1 and 3. Note that *Pegoscapus gemellus* A, marked with (**), is associated with two fig species. *P. gemellus* C was not found on receptive individuals of other *Ficus* species than the species it emerged from, so no estimates were possible here. Significant differences are indicated with a line, and all other comparisons are not significant. Confidence intervals can be found in Table 4.

As summarized in Figure 1 we expected that *Pegoscapus* pollinators would be trapped most often on trees belonging to the fig species they emerged from, but also with some frequency on other receptive *Urostigma* trees as well as at vegetative *Urostigma* trees due to overlap in volatile bouquets. The bootstrap estimates for six *Pegoscapus* pollinator species at receptive and vegetative trees that either do or do not belong to the species from which they emerged are plotted in Figure 5. For each *Pegoscapus* species, highest numbers were estimated for receptive trees of the fig species from which they emerged and lowest for vegetative trees belonging to another *Urostigma* species (Figure 5). As expected, intermediate estimates of pollinators were found at vegetative hosts belonging to the fig species from which they emerged as well as at receptive fig trees belonging to other *Ficus* species. While the same trend is observed for all wasp species, only some of the differences were statistically significant (Figure 5 and Table 4).

**TABLE 4:** 95% confidence intervals of the estimated numbers of pollinator at receptive or vegetative trees belonging either species from which it either emerged or not. Since most confidence intervals start at zero most estimates do not significantly differ.

Tree type	*Pegoscapus gemellus* A	*Pegoscapus gemellus* B	*Pegoscapus gemellus* C	*Pegoscapus hoffmeyeri* A	*Pegoscapus hoffmeyeri* B	*Pegoscapus tonduzi*
Receptive host species from which it emerged	0–54.9	0.825–1001.55	0–15.9	0.245–10.675	0.665–24.15	19.0–770
Receptive other *Urostigma* hosts	0–2.32	0–1.35	NA	0–1.65	0–3.3	0–5.565
Vegetative host species from which it emerged	0–0.075	0–1.825	0–1.475	0–1.353333	0–2.9025	0–10.615
Vegetative other *Urostigma* host	0–0.0225	0–0.02	0–0.02667	0–0.053333	0–0.165	0–0.06

## 4 DISCUSSION

With some exceptions, pollinating wasps exhibit high fig host specificity, though increasingly host sharing and host switching have been documented (Moe et al., 2011; Molbo et al., 2003; Satler et al., 2019). Host switching and sharing are consistent with genetic data indicating that hybridization and introgression occur over ecological time scales and that these processes have occurred throughout the history of *Ficus* (Gardner et al., 2023; Satler et al., 2022; Wang, Zhang, et al., 2021). Host specificity will depend on fig host recognition and therefore identifying cues used by pollinators to identify an appropriate host within their ecological context is important (Bronstein, 1987; Compton, 1993). Many previous studies have suggested that there is an “aerial pool” of pollinator wasps from which some locate their receptive hosts (e.g., Compton et al., 1988; Nason et al., 1996). These studies, in turn, have motivated other studies that have focused on assessing pollinator attraction to detached receptive syconia, and volatile blends produced by these (Grison‐Pigé et al., 2002, van Noort et al., 1989, Wang et al., 2013; Wang, Yang, et al., 2021; Ware & Compton, 1994a). This has established the role of chemical signals produced by receptive fig syconia in attracting wasp pollinators but does not give ecological context or describe how they find hosts in nature. Therefore, we put out sticky traps at non‐*Ficus* trees and *Ficus* trees of 13 fig species and across two developmental phases (receptive and vegetative). We did this in a diverse tropical fig community for which there exists extensive genetic data on both host fig species and pollinating wasp species. Specifically, previous studies suggest that across wasp species there is a continuum of specificity in which some pollinators species are very specific to certain fig host species, and in some cases, what appears to be the same wasp species are shared between host fig species (Cook & Segar, 2010; Machado et al., 2005; Molbo et al., 2003). It appears that host fig species that share wasp species often hybridize (Satler et al., 2022). We found: (1) wasps were only very rarely captured at non‐*Ficus* trees; (2) nonetheless, at four of the *Ficus* species pollinators were trapped often at vegetative tree individuals belonging to the *Ficus* species from which that wasp species routinely emerges; (3) overwhelmingly, wasp individuals were captured at receptive host trees that correspond to their usual fig host species.

Our results support for some fig species the hypothesis that volatiles produced by other than the receptive syconia promote pollinator attraction (Figure 3). We note that pollinator individuals that arrive at vegetative fig trees (Figures 2 and 3; also see Bronstein, 1987; Compton, 1993; Ware & Compton, 1994a) make costly mistakes since no reproduction is possible, given their short lives and usually great distances between conspecific *Ficus* trees, and female fig wasps will have little time left to search for a receptive host. Therefore, selection should favor female fig wasps that cue in on volatile signals that are only produced by the host tree when receptive. However, the signal from vegetative individual trees to which they respond seem to be attractive and even sufficient to distinguish from the species from which they emerged from other fig species (Figures 4 and 5). Like many other insects, for example, parasitoids, fig wasps may face a “reliability‐detectability problem” (Vet & Dicke, 1992) in which an individual pollinator is able to detect an individual host of fig species from which it emerged from a distance, but must be relatively close to determine whether or not that host bears receptive syconia. Fig pollinators may therefore respond to different cues during different phases of host selection. Fig leaves would likely provide a large emission surface area for at least part of the pollinator‐attracting signal with the potential to signal over large distance. The syconia are likely to produce additional volatile cues that reliably confer both the species identity and the developmental phase of the tree over a shorter distance. After arrival on the host tree a combination of volatile and contact cues likely guide a pollinator to the ostiole of a receptive syconium. We note a study reporting fig pollinators arriving to monoecious *Ficus burtt‐davyi* to land on leaves after which they started searching for, and investigating syconia (Ware & Compton, 1994b), which is consistent with the suggested mechanism outlined above.

To our knowledge, no studies have directly investigated the pollinator‐attracting potential of vegetative fig parts. However, what we have found within this Panamanian *Ficus* community is consistent with other examples. For example, synergy between vegetative and floral volatiles can be found in the pollinating hawkmoth *Manduca sexta* which shows a stronger response to floral volatiles if they are presented against a conspecific leaf volatile background (Kárpáti et al., 2013). Similary, the European dwarf palm (*Chamaerops humilis*) has been shown to emit pollinator‐attracting compounds from its leaves and not from its flowers (Dufaÿ et al., 2003). We hypothesize that signaling by leaves is more likely to be found in monoecious than in dioecious fig species. Monoecious fig species generally have lower densities, and their pollinator species are thought to disperse above the canopy as opposed to pollinator species of dioecious figs that are thought to disperse within the forest (Compton et al., 2000; Harrison, 2003; Harrison & Rasplus, 2006; Yang et al., 2015). A longer distance dispersal is inferred for pollinator species of monoecious fig species as well (Ahmed et al., 2009; Nason et al., 1998).

In conjunction with detailed ecological studies that document patterns of wasp presence and abundance with respect to their usual host and its developmental phase, more comparative chemical work is also needed. Studies on volatile bouquets emitted by fig trees often focus on syconia, the fig reproductive structures (Chen et al., 2009; Cornille et al., 2012; Grison‐Pigé et al., 2002; Proffit & Johnson, 2009; Wang et al., 2013, 2016). From these studies, we know that figs generally emit common plant volatiles, and that they share many of them across the entire genus (Borges et al., 2008; Grison‐Pigé et al., 2002; Proffit & Johnson, 2009). There are a few examples showing that the volatile bouquet of fig leaves partly overlap with those from syconia (Borges et al., 2008; Conchou et al., 2014; Song et al., 2001).

### 4.1 The balance of specificity and occasional “mistakes”

Most pollinator individuals are present at trees belonging to the fig species from which they emerged even when these trees are vegetative (Figures 3, 4, 5). But at the same time pollinator individuals do frequently arrive at closely related trees belonging to species in which they did not develop (Figures 4 and 5) Fig wasps may have a lower probability to produce offspring in syconia of a fig species which from which they usually do not emerge, for example, due to reduced ability to enter syconia through the ostioles or a lower survival rate of developing offspring (Ghana et al., 2015; Moe & Weiblen, 2012; Wang et al., 2013). Therefore, selection should favor fig pollinators that cue in on volatile signals that are only produced by the fig species from which they emerged. Nevertheless, pollinator fitness on other hosts will not be zero in all cases (Yang et al., 2012). This may be why fig pollinators are sometimes found on other fig species (Figure 4; but also see Bronstein, 1987; Wang et al., 2016; Ware & Compton, 1994a). If a pollinator individual, during her short lifespan, does not locate a trees with receptive syconia belonging to the species from which she emerged, she may settle for a suboptimal choice if it provides even a small chance of producing some offspring. An easily testable hypothesis, predicted by dynamic optimal foraging models (Mangel, 1992), that would provide support for this idea is that the host preference of pollinator individuals broadens as they age.

Behavioral “mistakes” by pollinators individuals can be potentially accounted for by high similarity in volatile bouquets between fig species (Cornille et al., 2012; Wang et al., 2016). For plant–pollinator relationships an increasing number of studies suggest that similar volatiles attract similar pollinators (Burkle & Runyon, 2019; Hetherington‐Rauth & Ramírez, 2016; Huang et al., 2015; Stökl et al., 2005). As with other plant groups, fig volatiles may be phylogenetically constrained (Joffard et al., 2020; Schwery et al., 2022). The degree to which different levels of phylogenetic distance predicts similarity of chemical cues should correspond to particular pollinator species being more likely to shift within fig sections or subgenera than between them (Cook & Segar, 2010). In our field site, we tentatively predict that if we expanded detailed sampling to *Pharmacosycea* figs and their *Tetrapus* pollinators, we expect to trap *Pegoscapus* pollinators more frequently at *Urostigma* figs than at *Pharmacosycea* figs, and the opposite for *Tetrapus* fig wasps (Figure 1). We note that the idea that shifts to new hosts are mediated by the chemical similarity between old and new hosts was already postulated in 1964 (Ehrlich & Raven, 1964), and supporting evidence has been found for many herbivorous insect groups (Becerra, 1997; Erbilgin et al., 2014; Murphy & Feeny, 2006; Rigsby et al., 2017).

Figs do not seem to have strong post‐zygotic isolating mechanisms, based on studies on natural and artificial hybrids showing that these produce viable seeds that develop well (Condit, 1950; Moe & Weiblen, 2012; Ramirez, 1986, 1994; Wang et al., 2013), although in one study syconia receiving pollinators with heterospecific pollen were more likely to abort (Wang et al., 2013). Recently, a backcross individual from a hybrid was found in Central Panama (Satler et al., 2022). Host‐choice errors or genuine flexibility in host choice by fig pollinators may lead to hybridization of *Ficus* species (Gardner et al., 2023; Satler et al., 2022; Wang, Yang, et al., 2021; Wang, Zhang, et al., 2021). Offspring developing in another host fig species may imprint on the cues of the new host species, and this could lead to a population establishing on this new host species making the host‐shift permanent as has been shown in other animals (Gowri et al., 2019; Remy, 2010; van Emden, 2015; Zhang et al., 2007).

The response of fig pollinators to host‐specific signals within the volatile bouquets produced by the species in which they developed is thought to play a major role in host specificity (Cornille et al., 2012; Grison‐Pigé et al., 2002; Herre et al., 2008; Wang et al., 2013, 2016; Wang, Yang, et al., 2021). Fig pollinators can locate and find a receptive individual of the species from which they emerged within the suite of volatile bouquets they encounter in the rainforest (Bronstein, 1987; van Noort et al., 1989; Ware & Compton, 1994b). Our findings provide ecological context within which to frame studies on how different factors interact in pollinator attraction. We found modest support for our two, non‐mutually exclusive, hypotheses which combined could explain how pollinator‐attraction by fig trees could balance both specificity as well as occasional mistakes. Future studies on host choice should integrate how chemical signals, notably not only from inflorescences but also from vegetative tissues, operate at the community level, and different phylogenetic levels. We believe this will be a very fruitful way forward toward explaining how the host specificity of fig pollinators relates to genetic diversification or isolation which, in turn, are expected to affect opportunities for speciation.

## AUTHOR CONTRIBUTIONS

**Aafke Oldenbeuving:** Conceptualization (equal); data curation (equal); formal analysis (equal); funding acquisition (equal); investigation (equal); methodology (equal); project administration (lead); resources (equal); validation (equal); visualization (lead); writing – original draft (lead). **Adalberto Gómez‐Zúniga:** Data curation (equal); investigation (equal); methodology (equal). **Ximena Florez‐Buitrago:** Data curation (supporting); investigation (equal); writing – original draft (supporting). **Ana M. Guiterrez‐Zuluaga:** Investigation (equal); writing – review and editing (supporting). **Carlos A. Machado:** Formal analysis (equal); investigation (equal); methodology (equal); writing – review and editing (equal). **Tom J. M. Van Dooren:** Formal analysis (equal); investigation (equal); methodology (equal); visualization (supporting); writing – review and editing (equal). **Jacques van Alphen:** Conceptualization (equal); funding acquisition (equal); supervision (equal); writing – review and editing (equal). **Jacobus C. Biesmeijer:** Conceptualization (equal); funding acquisition (equal); resources (equal); supervision (equal); writing – review and editing (equal). **Edward Allen Herre:** Conceptualization (equal); methodology (equal); project administration (equal); resources (equal); supervision (equal); writing – review and editing (equal).

## FUNDING INFORMATION

This research was funded by a NWO doctoral grant for teachers awarded to Aafke Oldenbeuving, and by the KNAW Ecology Fund also awarded to Aafke Oldenbeuving.

## Supporting information


Data S1.

## Data Availability

The data supporting our findings and R scripts are openly available via Dryad; https://doi.org/10.5061/dryad.6m905qg5j (submitted). COI sequences have been deposited in GenBank with accession numbers OR288903‐OR289513.
