# Supplementary material for: Field sampling of fig pollinator wasps across host species and host developmental phase: Implications for host recognition and specificity
Source: Ecol Evol. 2023 Sep 11;13(9):e10501. doi: 10.1002/ece3.10501 (PMC10495548; doi:10.1002/ece3.10501)
Supplement: Supplementary file 1 — Data S1. [file ECE3-13-e10501-s001.zip › SupInfo.docx]

**SUPPLEMENTAL MATERIAL**

| host species | pollinator code in phylogeny | pollinator genus |
| --- | --- | --- |
| *Ficus paraensis* | W1, Fparn | *Pegoscapus* |
| *Ficus obtusifolia* | W2 | *Pegoscapus* |
| *Ficus citrifolia* | W3 | *Pegoscapus* |
| *Ficus popenoei* | W4 | *Pegoscapus* |
| *Ficus nymphaefolia* | W5 | *Pegoscapus* |
| *Ficus trigonata (crocata)* | W6, F.trig | *Pegoscapus* |
| *Ficus triangle* | W7 | *Pegoscapus* |
| *Ficus dugandii* | W12 | *Pegoscapus* |
| *Ficus turbinata* | W13 | *Pegoscapus* |
| *Ficus pertusa* | W14 | *Pegoscapus* |
| *Ficus bullenei* | W15 | *Pegoscapus* |
| *Ficus colubrinae* | W16 | *Pegoscapus* |
| *Ficus pertusa* | W17 | *Pegoscapus* |
| *Ficus costaricana (americana)* | W18 | *Pegoscapus* |
| *Ficus velutina* | W19 | *Pegoscapus* |
| *Ficus flaevigata* | W20 | *Pegoscapus* |
| *Ficus gomezii* | F.gome | *Pegoscapus* |
| *Ficus davidii* | F.davidi | *Pegoscapus* |
| *Ficus petiolaris* | Fpetiolaris | *Pegoscapus* |
| *Ficus yoponensis* | W8 | *Tetrapus* |
| *Ficus maxima* | W9 | *Tetrapus* |
| *Ficus insipida* | W10 | *Tetrapus* |
| *Ficus glabrata* | W11 | *Tetrapus* |

table S1. Codes for the reference samples used for the neighbour joining trees in figures S1 – S11.

Figure S1. Neighbor Joining Tree showing relationships among COI sequences from individual wasps collected from multiple fig species from the Neotropics. Outgroup sequences are *Tetrapus spp*., and ingroup sequences are *Pegoscapus spp*. Individuals collected from *Ficus bullenei* for this study are highlighted in red. Names for each taxon in the phylogeny start with a code that either reflects its host species (see table S1) or their GenBank accession number and host (e.g. AB308339_Fpert is accession AB308339 associated with *F. pertusa*).

Figure S2. Neighbor Joining Tree showing relationships among COI sequences from individual wasps collected from multiple fig species from the Neotropics. Outgroup sequences are *Tetrapus spp*., and ingroup sequences are *Pegoscapus spp*. Individuals collected from *Ficus citrifolia* for this study are highlighted in red. Names for each taxon in the phylogeny start with a code that either reflects its host species (see table S1) or their GenBank accession number and host (e.g. AB308339_Fpert is accession AB308339 associated with *F. pertusa*).

Figure S3. Neighbor Joining Tree showing relationships among COI sequences from individual wasps collected from multiple fig species from the Neotropics. Outgroup sequences are *Tetrapus spp*., and ingroup sequences are *Pegoscapus spp*. Individuals collected from *Ficus colubrinae* for this study are highlighted in red. Names for each taxon in the phylogeny start with a code that either reflects its host species (see table S1) or their GenBank accession number and host (e.g. AB308339_Fpert is accession AB308339 associated with *F. pertusa*).

Figure S4. Neighbor Joining Tree showing relationships among COI sequences from individual wasps collected from multiple fig species from the Neotropics. Outgroup sequences are *Tetrapus spp*., and ingroup sequences are *Pegoscapus spp*. Individuals collected from *Ficus dugandiii* for this study are highlighted in red. Names for each taxon in the phylogeny start with a code that either reflects its host species (see table S1) or their GenBank accession number and host (e.g. AB308339_Fpert is accession AB308339 associated with *F. pertusa*).

Figure S5. Neighbor Joining Tree showing relationships among COI sequences from individual wasps collected from multiple fig species from the Neotropics. Outgroup sequences are *Tetrapus spp*., and ingroup sequences are *Pegoscapus spp*. Individuals collected from *Ficus near trigonata* for this study are highlighted in red. Names for each taxon in the phylogeny start with a code that either reflects its host species (see table S1) or their GenBank accession number and host (e.g. AB308339_Fpert is accession AB308339 associated with *F. pertusa*).

Figure S6. Neighbor Joining Tree showing relationships among COI sequences from individual wasps collected from multiple fig species from the Neotropics. Outgroup sequences are *Tetrapus spp*., and ingroup sequences are *Pegoscapus spp*. Individuals collected from *Ficus obtusifolia* for this study are highlighted in red. Names for each taxon in the phylogeny start with a code that either reflects its host species (see table S1) or their GenBank accession number and host (e.g. AB308339_Fpert is accession AB308339 associated with *F. pertusa*).

Figure S7. Neighbor Joining Tree showing relationships among COI sequences from individual wasps collected from multiple fig species from the Neotropics. Outgroup sequences are *Tetrapus spp*., and ingroup sequences are *Pegoscapus spp*. Individuals collected from *Ficus paraensis* for this study are highlighted in red. Names for each taxon in the phylogeny start with a code that either reflects its host species (see table S1) or their GenBank accession number and host (e.g. AB308339_Fpert is accession AB308339 associated with *F. pertusa*).

Figure S8. Neighbor Joining Tree showing relationships among COI sequences from individual wasps collected from multiple fig species from the Neotropics. Outgroup sequences are *Tetrapus spp*., and ingroup sequences are *Pegoscapus spp*. Individuals collected from *Ficus perforata* for this study are highlighted in red. Names for each taxon in the phylogeny start with a code that either reflects its host species (see table S1) or their GenBank accession number and host (e.g. AB308339_Fpert is accession AB308339 associated with *F. pertusa*).

Figure S9. Neighbor Joining Tree showing relationships among COI sequences from individual wasps collected from multiple fig species from the Neotropics. Outgroup sequences are *Tetrapus spp*., and ingroup sequences are *Pegoscapus spp*. Individuals collected from *Ficus pertusa* for this study are highlighted in red. Names for each taxon in the phylogeny start with a code that either reflects its host species (see table S1) or their GenBank accession number and host (e.g. AB308339_Fpert is accession AB308339 associated with *F. pertusa*).

Figure S10. Neighbor Joining Tree showing relationships among COI sequences from individual wasps collected from multiple fig species from the Neotropics. Outgroup sequences are *Tetrapus spp*., and ingroup sequences are *Pegoscapus spp*. Individuals collected from *Ficus popenoei* for this study are highlighted in red. Names for each taxon in the phylogeny start with a code that either reflects its host species (see table S1) or their GenBank accession number and host (e.g. AB308339_Fpert is accession AB308339 associated with *F. pertusa*).

Figure S11. Neighbor Joining Tree showing relationships among COI sequences from individual wasps collected from multiple fig species from the Neotropics. Outgroup sequences are *Tetrapus spp*., and ingroup sequences are *Pegoscapus spp*. Individuals collected from *Ficus trigonata* for this study are highlighted in red. Names for each taxon in the phylogeny start with a code that either reflects its host species (see table S1) or their GenBank accession number and host (e.g. AB308339_Fpert is accession AB308339 associated with *F. pertusa*).
