# Supplementary material for: Field sampling of fig pollinator wasps across host species and host developmental phase: Implications for host recognition and specificity
Source: Ecol Evol. 2023 Sep 11;13(9):e10501. doi: 10.1002/ece3.10501 (PMC10495548; doi:10.1002/ece3.10501)
Supplement: Supplementary file 1 — Data S1. [file ECE3-13-e10501-s001.zip › SuppMaterial R script barcoding data.rtf]

## AUTHORS Tom Van Dooren and Aafke Oldenbeuving## version March 3rd 2023## aafke.oldenbeuving@naturalis.nl#load librarieslibrary(MASS) # for stepaiclibrary(lme4)#prepare data#everything as factorsdatabar$correct<-as.factor(databar$correct)#replace 15 "?", unknown species, for "0" meaning not-specificdatabar["correct"][databar["correct"] == "?"] <- "0"# question is; are pollinator more often 'correct' in the receptive phase# mixed effect model, random species effect#first modelglmmmax<-glmer(correct~HOST_SP*PHASE+(1|pollID),family=binomial,data=databar)# failure to converge#second modelglmmax<-glm(correct~HOST_SP*PHASE+pollID,family=binomial,data=databar)#Warning message:#  glm.fit: fitted probabilities numerically 0 or 1 occurred ### is this warning message problematic?summary(glmmax) #only two significant parametersglmsel1<-stepAIC(glmmax) # drop1(glmsel1,test="Chisq") # # third modelglm2<-glm(correct~HOST_SP*PHASE,family=binomial,data=databar)drop1(glm2,test="Chisq")summary(glm2)glm3<-glm(correct~HOST_SP+PHASE,family=binomial,data=databar)drop1(glm3,test="Chisq")summary(glm3)confint(glm3)#2.5 %     97.5 %#  (Intercept)   1.14091560  2.5931948 (***)#HOST_SPCIT    2.49157139  7.0278277 (***)#HOST_SPCOL   -2.02559696  0.7140643 #HOST_SPDUG   -0.27547766  4.4174238#HOST_SPOBT    1.87131050  3.5495851 (***)#HOST_SPPAR   -1.57005360  0.8964583#HOST_SPPOP   -0.09009836  1.3181774#HOST_SPPRF   -1.38835999  0.7921956#HOST_SPPRT   -3.29370878 -0.3704670#HOST_SPTRG   -0.76796665  3.9648593#HOST_SPTRN    0.03777367  2.6539538 (*)#PHASEsterile -3.31682738 -1.8715634 (***)######### RERUN WITHOUT LOPESI-POLLINATORS AT BOHIO44 ########## import data file "BARC_Carlos" sheet minus_BULLlopesi as databar2#prepare data#everything as factorsdatabar2$correct<-as.factor(databar2$correct)#replace 15 "?", unknown species, for "0" meaning not-specificdatabar2["correct"][databar2["correct"] == "?"] <- "0"# using the same model as above# not going through all models...glm32<-glm(correct~HOST_SP+PHASE,family=binomial,data=databar2)drop1(glm32,test="Chisq")summary(glm32)#SPECIES and PHASE beide signifcant dus blijven in het model??# AIC van model met alleen PHASE is wel lager??# (Intercept) and PHASEsterile zijn significantconfint(glm32)#2.5 %      97.5 %#  (Intercept)   1.8528187  3.65848196#HOST_SPCIT    1.0592428  5.73883117#HOST_SPCOL   -3.0191265 -0.07493120#HOST_SPDUG   -1.2492381  3.58260909#HOST_SPOBT    0.3017864  2.31081370#HOST_SPPAR   -2.5761753  0.11682553#HOST_SPPOP   -1.5524608  0.21114065#HOST_SPPRF   -2.4481352 -0.05517537#HOST_SPPRT   -4.2662439 -1.15630059#HOST_SPTRG   -1.7391360  3.12992166#HOST_SPTRN   -1.3209414  1.45498935#PHASEsterile -2.8000629 -1.31295520
