# Supplementary material for: Field sampling of fig pollinator wasps across host species and host developmental phase: Implications for host recognition and specificity
Source: Ecol Evol. 2023 Sep 11;13(9):e10501. doi: 10.1002/ece3.10501 (PMC10495548; doi:10.1002/ece3.10501)
Supplement: Supplementary file 1 — Data S1. [file ECE3-13-e10501-s001.zip › SuppMaterial R script bootstrapping.rtf]

#AUTHORS Tom Van Dooren and Aafke Oldenbeuving#version March 3th 2023#aafke.oldenbeuving@naturalis.nl# bootstrap for counts and proportions# select a data subset of counts and the corresponding one for proportions####### first import count and barcoding data ##########”TRAP DATA” sheet named bootstrapR and I named it datac#”BARCODES edited selection sheet named bootstrapR and I named it databar#for each pollinator species I added an extra variable to the data of which the#levels are fl_host, fl_nonhost, veg_host, AND veg_nonhostlibrary(ggplot2)library(plyr)library(ggsignif)#square-root transformation formulass <- scales::trans_new("ssqrt", transform=function(x) sign(x)*sqrt(abs(x)), inverse=function(x) sign(x)*x^2)######################  bootstraps of counts from traps and proportions from barcodes ######AAFKE: for the counts I used this script #that I found online and I modified it a bit# answer 6 from https://stackoverflow.com/questions/23831711/sample-n-random-rows-per-group-in-a-dataframe# This solution splits the data.frame into groups # (fl_host, fl_nonhost, veg_host, AND veg_nonhost)# Then it samples 200 rows from each group with replacement.# Then I merged the list of data.frames into one dataframe.# I repeat it for each species; NOTE groups are different for each species##### estimate for Pegoscapus gemellus A ######datac_gemA <- lapply(split(datac, datac$for_gemA),                     function(subdf) subdf[sample(1:nrow(subdf), 200, replace = TRUE),])bc_gemA<-rbind(datac_gemA$fl_host,datac_gemA$fl_nonhost,               datac_gemA$veg_host,datac_gemA$veg_nonhost)#bc means “bootstrap count”data.frame(bc_gemA)View(bc_gemA)#and then I plotted these resampled counts (800 in total, 200 per group)as.factor(bc_gemA$for_gemA)ggplot(bc_gemA, aes(y=wpd,fill=for_gemA))+  geom_boxplot()##### proportions ######to resample the barcoding data to get estimates of the proportions # I used the script of TomVanDooren but than on the groups (levels of factor) I added# I do the same steps 4 times for each group# (fl_host, fl_nonhost, veg_host, AND veg_nonhost)### 1 proportions for flowering hosts of gemAsubdatprop_fl_host_gemA<-subset(databar,for_gemA=="fl_host")propresamples_fl_host_gemA<-list()for(i in 1:200)propresamples_fl_host_gemA[[i]]<-sample(factor(subdatprop_fl_host_gemA$pollID),200,replace=T)# convert into proportions for gem A hostsproper<-function(x){tab<-table(x);tab/sum(tab)}reprops_fl_host_gemA<-lapply(propresamples_fl_host_gemA,proper)View(reprops_fl_host_gemA)#a made a data.frame with probablilities for gemA hosts#since I don’t really know how to used these lists of dataframesprop_fl_host_gemA <- data.frame(do.call("rbind",reprops_fl_host_gemA))### 2 proportions for flowering non-hosts of gemAsubdatprop_fl_nonhost_gemA <-subset(databar,for_gemA=="fl_nonhost")propresamples_fl_nonhost_gemA<-list()for(i in 1:200)propresamples_fl_nonhost_gemA[[i]]<-sample(factor(subdatprop_fl_nonhost_gemA$pollID),200,replace=T)# convert into proportions for gemA non-hostsproper<-function(x){tab<-table(x);tab/sum(tab)}reprops_fl_nonhost_gemA<-lapply(propresamples_fl_nonhost_gemA,proper)View(reprops_fl_nonhost_gemA)#getting a data.frame with probablilities gemA non-hostsprop_fl_nonhost_gemA <- data.frame(do.call("rbind",reprops_fl_nonhost_gemA))dim(prop_fl_nonhost_gemA)####################### 3 proportions for vegetative hosts of gemAsubdatprop_veg_host_gemA<-subset(databar,for_gemA=="veg_host")propresamples_veg_host_gemA<-list()for(i in 1:200)propresamples_veg_host_gemA[[i]]<-sample(factor(subdatprop_veg_host_gemA$pollID),200,replace=T)View(propresamples_veg_host_gemA)# convert into proportions for gemA non-hostsproper<-function(x){tab<-table(x);tab/sum(tab)}reprops_veg_host_gemA<-lapply(propresamples_veg_host_gemA,proper)View(reprops_veg_host_gemA)#getting a data.frame with probablilities gemA non-hostsprop_veg_host_gemA <- data.frame(do.call("rbind",reprops_veg_host_gemA))View(prop_veg_host_gemA$P_gemA)####################### 4 proportions for vegetative non-hosts of gemAsubdatprop_veg_nonhost_gemA<-subset(databar,for_gemA=="veg_nonhost")propresamples_veg_nonhost_gemA<-list()for(i in 1:200)propresamples_veg_nonhost_gemA[[i]]<-sample(factor(subdatprop_veg_nonhost_gemA$pollID),200,replace=T)# convert into proportions for gemA non-hostsproper<-function(x){tab<-table(x);tab/sum(tab)}reprops_veg_nonhost_gemA<-lapply(propresamples_veg_nonhost_gemA,proper)View(reprops_veg_nonhost_gemA)#getting a data.frame with probablilities gemA non-hostsprop_veg_nonhost_gemA <- data.frame(do.call("rbind",reprops_veg_nonhost_gemA))View(prop_veg_nonhost_gemA$P_gemA)###################and then I made dataframe of the estimated proportion of the 4 groupsreprops_hosts_gemA <- rbind.fill(prop_fl_host_gemA,prop_fl_nonhost_gemA,                                 prop_veg_host_gemA,prop_veg_nonhost_gemA)#and then I collected the data to multiply the counts and the proportionslabels <- c("fl_host","fl_nonhost","veg_host","veg_nonhost")type_gemA <- rep(labels,each=200)bc_gemA <- as.numeric(bc_gemA$wpd)bb_gemA <- as.numeric(reprops_hosts_gemA$P_gemA)#multiplying bootstrapped counts and bootstrapped proportionsest_gemA <- bb_gemA*bc_gemA#make a dataframe againdf_est_gemA <- data.frame(cbind(type_gemA,                                bc_gemA,                                bb_gemA,                                est_gemA))#plot the estimates just to check#needed to get numeric datadf_est_gemA$est_gemA <- as.numeric(as.character(df_est_gemA$est_gemA))#note that some values may not be numeric causing problems#plot_gemA<-ggplot(df_est_gemA, aes(type_gemA,est_gemA))+  geom_boxplot()+  theme_classic()+  scale_y_continuous(trans = ss)+  labs(x=NULL,       y="estimated number of Pegoscapus gemellus A per 24h\n       (square-root transformed)")+  scale_x_discrete(limits = c('fl_host','fl_nonhost',                              'veg_host','veg_nonhost'),                   labels = c('receptive\nassociated\nUrostigma fig',                              'receptive\nother\nUrostigma fig',                              'vegetative\nassociated\nUrostigma fig',                              'vegetative\nother\nUrostigma fig'))#trying to sort the estimates per group#first the sorting didn’t work out well, but I found this ‘trick’ below#https://stackoverflow.com/questions/29399518/order-function-in-r-is-not-ordering-correctly/29401459df_est_gemA$est_gemA <- as.numeric(as.character(df_est_gemA$est_gemA))############# getting confidence intervals #########################flowering gemA hosts#subset estimates for flowering gemA hostsfl_host_est_gemA <- subset(df_est_gemA,type_gemA=="fl_host")fl_host_est_gemA<-  fl_host_est_gemA[order(fl_host_est_gemA$est_gemA),]View(fl_host_est_gemA)#calculating confidence intervals for flowering hostsfl_host_est_gemA$est_gemA[6] #0fl_host_est_gemA$est_gemA[195] #54.855####flowering gemA non-hosts#subset estimates for flowering gemA non-hostsfl_nonhost_est_gemA <- subset(df_est_gemA,type_gemA=="fl_nonhost")fl_nonhost_est_gemA<-  fl_nonhost_est_gemA[order(fl_nonhost_est_gemA$est_gemA),]#calculating confidence intervals for flowering non-hostsfl_nonhost_est_gemA$est_gemA[6] #0fl_nonhost_est_gemA$est_gemA[195] #2.32####vegetative gemA hosts#subset estimates for vegetative gemA hostsveg_host_est_gemA <- subset(df_est_gemA,type_gemA=="veg_host")veg_host_est_gemA<-  veg_host_est_gemA[order(veg_host_est_gemA$est_gemA),]#calculating confidence intervals for vegetative hostsveg_host_est_gemA$est_gemA[6] #0veg_host_est_gemA$est_gemA[195] #0.075####vegetative gemA non-hosts#subset estimates for vegetative gemA non-hostsveg_nonhost_est_gemA <- subset(df_est_gemA,type_gemA=="veg_nonhost")veg_nonhost_est_gemA<-  veg_nonhost_est_gemA[order(veg_nonhost_est_gemA$est_gemA),]#calculating confidence intervals for vegetative non-hostsveg_nonhost_est_gemA$est_gemA[6] #0veg_nonhost_est_gemA$est_gemA[195] #0.0225#################################################### estimate for Pegoscapus gemellus B #####################################################datac_gemB <- lapply(split(datac, datac$for_gemB),                     function(subdf) subdf[sample(1:nrow(subdf), 200, replace = TRUE),])bc_gemB<-rbind(datac_gemB$fl_host,datac_gemB$fl_nonhost,               datac_gemB$veg_host,datac_gemB$veg_nonhost)#bc means “bootstrap count”data.frame(bc_gemB)View(bc_gemB)#and then I plotted these resampled counts (800 in total, 200 per group)as.factor(bc_gemB$for_gemB)ggplot(bc_gemB, aes(y=wpd,fill=for_gemB))+  geom_boxplot()##### proportions ######to resample the barcoding data to get estimates of the proportions # I used the script of TomVanDooren but than on the groups (levels of factor) I added# I do the same steps 4 times for each group# (fl_host, fl_nonhost, veg_host, AND veg_nonhost)### 1 proportions for flowering hosts of gemBsubdatprop_fl_host_gemB<-subset(databar,for_gemB=="fl_host")propresamples_fl_host_gemB<-list()for(i in 1:200)propresamples_fl_host_gemB[[i]]<-sample(factor(subdatprop_fl_host_gemB$pollID),200,replace=T)# convert into proportions for gemB hostsproper<-function(x){tab<-table(x);tab/sum(tab)}reprops_fl_host_gemB<-lapply(propresamples_fl_host_gemB,proper)View(reprops_fl_host_gemB)#a made a data.frame with probablilities for gemB hosts#since I don’t really know how to used these lists of dataframesprop_fl_host_gemB <- data.frame(do.call("rbind",reprops_fl_host_gemB))### 2 proportions for flowering non-hosts of gemBsubdatprop_fl_nonhost_gemB <-subset(databar,for_gemB=="fl_nonhost")propresamples_fl_nonhost_gemB<-list()for(i in 1:200)propresamples_fl_nonhost_gemB[[i]]<-sample(factor(subdatprop_fl_nonhost_gemB$pollID),200,replace=T)# convert into proportions for gemB non-hostsproper<-function(x){tab<-table(x);tab/sum(tab)}reprops_fl_nonhost_gemB<-lapply(propresamples_fl_nonhost_gemB,proper)View(reprops_fl_nonhost_gemB)#getting a data.frame with probablilities gemB non-hostsprop_fl_nonhost_gemB <- data.frame(do.call("rbind",reprops_fl_nonhost_gemB))View(prop_fl_nonhost_gemB$P_gemB)####################### 3 proportions for vegetative hosts of gemBsubdatprop_veg_host_gemB<-subset(databar,for_gemB=="veg_host")propresamples_veg_host_gemB<-list()for(i in 1:200)propresamples_veg_host_gemB[[i]]<-sample(factor(subdatprop_veg_host_gemB$pollID),200,replace=T)# convert into proportions for gemB non-hostsproper<-function(x){tab<-table(x);tab/sum(tab)}reprops_veg_host_gemB<-lapply(propresamples_veg_host_gemB,proper)View(reprops_veg_host_gemB)#getting a data.frame with probablilities gemB non-hostsprop_veg_host_gemB <- data.frame(do.call("rbind",reprops_veg_host_gemB))View(prop_veg_host_gemB$P_gemB)####################### 4 proportions for vegetative non-hosts of gemBsubdatprop_veg_nonhost_gemB<-subset(databar,for_gemB=="veg_nonhost")propresamples_veg_nonhost_gemB<-list()for(i in 1:200)propresamples_veg_nonhost_gemB[[i]]<-sample(factor(subdatprop_veg_nonhost_gemB$pollID),200,replace=T)# convert into proportions for gemB non-hostsproper<-function(x){tab<-table(x);tab/sum(tab)}reprops_veg_nonhost_gemB<-lapply(propresamples_veg_nonhost_gemB,proper)View(reprops_veg_nonhost_gemB)#getting a data.frame with probablilities gemB non-hostsprop_veg_nonhost_gemB <- data.frame(do.call("rbind",reprops_veg_nonhost_gemB))View(prop_veg_nonhost_gemB$P_gemB)#and then I made dataframe of the estimated proportion of the 4 groupsreprops_hosts_gemB <- rbind.fill(prop_fl_host_gemB,prop_fl_nonhost_gemB,                                 prop_veg_host_gemB,prop_veg_nonhost_gemB)#and then I collected the data to multiply the counts and the proportionslabels <- c("fl_host","fl_nonhost","veg_host","veg_nonhost")type_gemB <- rep(labels,each=200)bc_gemB <- as.numeric(bc_gemB$wpd)bb_gemB <- as.numeric(reprops_hosts_gemB$P_gemB)#multiplying bootstrapped counts and bootstrapped proportionsest_gemB <- bb_gemB*bc_gemB#make a dataframe againdf_est_gemB <- data.frame(cbind(type_gemB,                                bc_gemB,                                bb_gemB,                                est_gemB))#plot the counts just to check#note that some values may not be numeric causing problemsdf_est_gemB$est_gemB <- as.numeric(as.character(df_est_gemB$est_gemB))#plot_gemB <-  ggplot(df_est_gemB, aes(type_gemB,est_gemB))+  geom_boxplot()+  theme_classic()+  scale_y_continuous(trans = ss)+  labs(x=NULL,       y="estimated number of Pegoscapus gemellus B per 24h\n       (square-root transformed)")+  scale_x_discrete(limits = c('fl_host','fl_nonhost',                              'veg_host','veg_nonhost'),                   labels = c('receptive\nassociated\nUrostigma fig',                              'receptive\nother\nUrostigma fig',                              'vegetative\nassociated\nUrostigma fig',                              'vegetative\nother\nUrostigma fig'))#trying to sort the estimates per group#first the sorting didn’t work out well, but I found this ‘trick’ below#https://stackoverflow.com/questions/29399518/order-function-in-r-is-not-ordering-correctly/29401459df_est_gemB$est_gemB <- as.numeric(as.character(df_est_gemB$est_gemB))############# getting confidence intervals #########################flowering gemB hosts#subset estimates for flowering gemB hostsfl_host_est_gemB <- subset(df_est_gemB,type_gemB=="fl_host")fl_host_est_gemB<-  fl_host_est_gemB[order(fl_host_est_gemB$est_gemB),]View(fl_host_est_gemB)#calculating confidence intervals for flowering hostsfl_host_est_gemB$est_gemB[6] #0.825fl_host_est_gemB$est_gemB[195] #1001.55####flowering gemB non-hosts#subset estimates for flowering gemB non-hostsfl_nonhost_est_gemB <- subset(df_est_gemB,type_gemB=="fl_nonhost")fl_nonhost_est_gemB<-  fl_nonhost_est_gemB[order(fl_nonhost_est_gemB$est_gemB),]#calculating confidence intervals for flowering non-hostsfl_nonhost_est_gemB$est_gemB[6] #0fl_nonhost_est_gemB$est_gemB[195] #1.35####vegetative gemB hosts#subset estimates for vegetative gemB hostsveg_host_est_gemB <- subset(df_est_gemB,type_gemB=="veg_host")veg_host_est_gemB<-  veg_host_est_gemB[order(veg_host_est_gemB$est_gemB),]#calculating confidence intervals for vegetative hostsveg_host_est_gemB$est_gemB[6] #0veg_host_est_gemB$est_gemB[195] #1.825####vegetative gemB non-hosts#subset estimates for vegetative gemB non-hostsveg_nonhost_est_gemB <- subset(df_est_gemB,type_gemB=="veg_nonhost")veg_nonhost_est_gemB<-  veg_nonhost_est_gemB[order(veg_nonhost_est_gemB$est_gemB),]#calculating confidence intervals for vegetative non-hostsveg_nonhost_est_gemB$est_gemB[6] #0veg_nonhost_est_gemB$est_gemB[195] #0.02#################################################### estimate for Pegoscapus gemellus C ########################################################## bootstrap count data  #####datac_gemC <- lapply(split(datac, datac$for_gemC),                     function(subdf) subdf[sample(1:nrow(subdf), 200, replace = TRUE),])bc_gemC<-rbind(datac_gemC$fl_host,datac_gemC$fl_nonhost,               datac_gemC$veg_host,datac_gemC$veg_nonhost)#bc means “bootstrap count”data.frame(bc_gemC)View(bc_gemC)#and then I plotted these resampled counts (800 in total, 200 per group)as.factor(bc_gemC$for_gemC)ggplot(bc_gemC, aes(y=wpd,fill=for_gemC))+  geom_boxplot()##### boottrap proportion data ######to resample the barcoding data to get estimates of the proportions # I used the script of TomVanDooren but than on the groups (levels of factor) I added# I do the same steps 4 times for each group# (fl_host, fl_nonhost, veg_host, AND veg_nonhost)### 1 proportions for flowering hosts of gemBsubdatprop_fl_host_gemC<-subset(databar,for_gemC=="fl_host")propresamples_fl_host_gemC<-list()for(i in 1:200)propresamples_fl_host_gemC[[i]]<-sample(factor(subdatprop_fl_host_gemC$pollID),200,replace=T)# convert into proportions for gemC hostsproper<-function(x){tab<-table(x);tab/sum(tab)}reprops_fl_host_gemC<-lapply(propresamples_fl_host_gemC,proper)View(reprops_fl_host_gemC)#a made a data.frame with probablilities for gemC hosts#since I don’t really know how to used these lists of dataframesprop_fl_host_gemC <- data.frame(do.call("rbind",reprops_fl_host_gemC))View(prop_fl_host_gemC)### 2 proportions for flowering non-hosts of gemCsubdatprop_fl_nonhost_gemC <-subset(databar,for_gemC=="fl_nonhost")propresamples_fl_nonhost_gemC<-list()for(i in 1:200)propresamples_fl_nonhost_gemC[[i]]<-sample(factor(subdatprop_fl_nonhost_gemC$pollID),200,replace=T)# convert into proportions for gemC non-hostsproper<-function(x){tab<-table(x);tab/sum(tab)}reprops_fl_nonhost_gemC<-lapply(propresamples_fl_nonhost_gemC,proper)View(reprops_fl_nonhost_gemC)#getting a data.frame with probablilities gemB non-hostsprop_fl_nonhost_gemC <- data.frame(do.call("rbind",reprops_fl_nonhost_gemC))View(prop_fl_nonhost_gemC$P_gemC)####################### 3 proportions for vegetative hosts of gemCsubdatprop_veg_host_gemC<-subset(databar,for_gemC=="veg_host")propresamples_veg_host_gemC<-list()for(i in 1:200)propresamples_veg_host_gemC[[i]]<-sample(factor(subdatprop_veg_host_gemC$pollID),200,replace=T)# convert into proportions for gemC non-hostsproper<-function(x){tab<-table(x);tab/sum(tab)}reprops_veg_host_gemC<-lapply(propresamples_veg_host_gemC,proper)View(reprops_veg_host_gemC)#getting a data.frame with probablilities gemC non-hostsprop_veg_host_gemC <- data.frame(do.call("rbind",reprops_veg_host_gemC))View(prop_veg_host_gemC$P_gemC)####################### proportions for vegetative non-hosts of gemCsubdatprop_veg_nonhost_gemC<-subset(databar,for_gemC=="veg_nonhost")propresamples_veg_nonhost_gemC<-list()for(i in 1:200)propresamples_veg_nonhost_gemC[[i]]<-sample(factor(subdatprop_veg_nonhost_gemC$pollID),200,replace=T)# convert into proportions for gemC non-hostsproper<-function(x){tab<-table(x);tab/sum(tab)}reprops_veg_nonhost_gemC<-lapply(propresamples_veg_nonhost_gemC,proper)View(reprops_veg_nonhost_gemC)#getting a data.frame with probablilities gemC non-hostsprop_veg_nonhost_gemC <- data.frame(do.call("rbind",reprops_veg_nonhost_gemC))View(prop_veg_nonhost_gemC$P_gemC)#and then I made dataframe of the estimated proportion of the 4 groupsreprops_hosts_gemC <- rbind.fill(prop_fl_host_gemC,prop_fl_nonhost_gemC,                                 prop_veg_host_gemC,prop_veg_nonhost_gemC)#and then I collected the data to multiply the counts and the proportionslabels <- c("fl_host","fl_nonhost","veg_host","veg_nonhost")type_gemC <- rep(labels,each=200)bc_gemC <- as.numeric(bc_gemC$wpd)bb_gemC <- as.numeric(reprops_hosts_gemC$P_gemC)#multiplying bootstrapped counts and bootstrapped proportionsest_gemC <- bb_gemC*bc_gemC#make a dataframe againdf_est_gemC <- data.frame(cbind(type_gemC,                                bc_gemC,                                bb_gemC,                                est_gemC))#note that some values may not be numeric causing problemsdf_est_gemC$est_gemC <- as.numeric(as.character(df_est_gemC$est_gemC))#plot_gemC<-  ggplot(df_est_gemC, aes(type_gemC,est_gemC))+  geom_boxplot()+  theme_classic()+  scale_y_continuous(trans = ss)+  labs(x=NULL,       y="estimated number of Pegoscapus gemellus C per 24h\n       (square-root transformed)")+  scale_x_discrete(limits = c('fl_host','fl_nonhost',                              'veg_host','veg_nonhost'),                   labels = c('receptive\nassociated\nUrostigma fig',                              'receptive\nother\nUrostigma fig',                              'vegetative\nassociated\nUrostigma fig',                              'vegetative\nother\nUrostigma fig'))#trying to sort the estimates per group#first the sorting didn’t work out well, but I found this ‘trick’ below#https://stackoverflow.com/questions/29399518/order-function-in-r-is-not-ordering-correctly/29401459df_est_gemC$est_gemC <- as.numeric(as.character(df_est_gemC$est_gemC))############# getting confidence intervals #########################flowering gemC hosts#subset estimates for flowering gemC hostsfl_host_est_gemC <- subset(df_est_gemC,type_gemC=="fl_host")fl_host_est_gemC<-  fl_host_est_gemC[order(fl_host_est_gemC$est_gemC),]View(fl_host_est_gemC)#calculating confidence intervals for flowering hostsfl_host_est_gemC$est_gemC[6] #0fl_host_est_gemC$est_gemC[195] #15.9####flowering gemC non-hosts#subset estimates for flowering gemC non-hostsfl_nonhost_est_gemC <- subset(df_est_gemC,type_gemC=="fl_nonhost")fl_nonhost_est_gemC<-  fl_nonhost_est_gemC[order(fl_nonhost_est_gemC$est_gemC),]#calculating confidence intervals for flowering non-hostsfl_nonhost_est_gemC$est_gemC[6] #NAfl_nonhost_est_gemC$est_gemC[195] #NA####vegetative gemC hosts#subset estimates for vegetative gemC hostsveg_host_est_gemC <- subset(df_est_gemC,type_gemC=="veg_host")veg_host_est_gemC<-  veg_host_est_gemC[order(veg_host_est_gemC$est_gemC),]#calculating confidence intervals for vegetative hostsveg_host_est_gemC$est_gemC[6] #0veg_host_est_gemC$est_gemC[195] #3.465####vegetative gemC non-hosts#subset estimates for vegetative gemC non-hostsveg_nonhost_est_gemC <- subset(df_est_gemC,type_gemC=="veg_nonhost")veg_nonhost_est_gemC<-  veg_nonhost_est_gemC[order(veg_nonhost_est_gemC$est_gemC),]#calculating confidence intervals for flowering non-hostsveg_nonhost_est_gemC$est_gemC[6] #0veg_nonhost_est_gemC$est_gemC[195] #0.02666667#################################################### estimate for Pegoscapus hoffmeyeri A ###########################################################  bootstrap count data ######AAFKE: I used this script that I found online and I modified it a bit# answer 6 from https://stackoverflow.com/questions/23831711/sample-n-random-rows-per-group-in-a-dataframe# This solution splits the data.frame into groups (fl_host, fl_nonhost, veg_host, AND veg_nonhost)# Then it samples 200 rows from each group with replacement.# Then I merged the list of data.frames into one dataframe.datac_hoffA <- lapply(split(datac, datac$for_hoffA),                      function(subdf) subdf[sample(1:nrow(subdf), 200, replace = TRUE),])bc_hoffA<-rbind(datac_hoffA$fl_host,datac_hoffA$fl_nonhost,                datac_hoffA$veg_host,datac_hoffA$veg_nonhost)#bc means “bootstrap count”data.frame(bc_hoffA)View(bc_hoffA)#and then I plotted these resampled counts (800 in total, 200 per group)as.factor(bc_hoffA$for_hoffA)ggplot(bc_hoffA, aes(y=wpd,fill=for_hoffA))+  geom_boxplot()##### bootstrap proportion data ######to resample the barcoding data to get estimates of the proportions # I used the script of TomVanDooren but than on the groups (levels of factor) I added# I do the same steps 4 times for each group# (fl_host, fl_nonhost, veg_host, AND veg_nonhost)### 1 proportions for flowering hosts of hoffAsubdatprop_fl_host_hoffA<-subset(databar,for_hoffA=="fl_host")propresamples_fl_host_hoffA<-list()for(i in 1:200)propresamples_fl_host_hoffA[[i]]<-sample(factor(subdatprop_fl_host_hoffA$pollID),200,replace=T)# convert into proportions for hoffA hostsproper<-function(x){tab<-table(x);tab/sum(tab)}reprops_fl_host_hoffA<-lapply(propresamples_fl_host_hoffA,proper)View(reprops_fl_host_hoffA)#a made a data.frame with probablilities for hoffA hosts#since I don’t really know how to used these lists of dataframesprop_fl_host_hoffA <- data.frame(do.call("rbind",reprops_fl_host_hoffA))### 2 proportions for flowering non-hosts of hoffAsubdatprop_fl_nonhost_hoffA <-subset(databar,for_hoffA=="fl_nonhost")propresamples_fl_nonhost_hoffA<-list()for(i in 1:200)propresamples_fl_nonhost_hoffA[[i]]<-sample(factor(subdatprop_fl_nonhost_hoffA$pollID),200,replace=T)# convert into proportions for hoffA non-hostsproper<-function(x){tab<-table(x);tab/sum(tab)}reprops_fl_nonhost_hoffA<-lapply(propresamples_fl_nonhost_hoffA,proper)View(reprops_fl_nonhost_hoffA)#getting a data.frame with probablilities hoffA non-hostsprop_fl_nonhost_hoffA <- data.frame(do.call("rbind",reprops_fl_nonhost_hoffA))View(prop_fl_nonhost_hoffA$P_hoffA)####################### 3 proportions for vegetative hosts of hoffAsubdatprop_veg_host_hoffA<-subset(databar,for_hoffA=="veg_host")propresamples_veg_host_hoffA<-list()for(i in 1:200)propresamples_veg_host_hoffA[[i]]<-sample(factor(subdatprop_veg_host_hoffA$pollID),200,replace=T)# convert into proportions for hoffA non-hostsproper<-function(x){tab<-table(x);tab/sum(tab)}reprops_veg_host_hoffA<-lapply(propresamples_veg_host_hoffA,proper)View(reprops_veg_host_hoffA)#getting a data.frame with probablilities hoffA non-hostsprop_veg_host_hoffA <- data.frame(do.call("rbind",reprops_veg_host_hoffA))View(prop_veg_host_hoffA$P_hoffA)####################### 4 proportions for vegetative non-hosts of hoffAsubdatprop_veg_nonhost_hoffA<-subset(databar,for_hoffA=="veg_nonhost")propresamples_veg_nonhost_hoffA<-list()for(i in 1:200)propresamples_veg_nonhost_hoffA[[i]]<-sample(factor(subdatprop_veg_nonhost_hoffA$pollID),200,replace=T)# convert into proportions for hoffA non-hostsproper<-function(x){tab<-table(x);tab/sum(tab)}reprops_veg_nonhost_hoffA<-lapply(propresamples_veg_nonhost_hoffA,proper)View(reprops_veg_nonhost_hoffA)#getting a data.frame with probablilities hoffA non-hostsprop_veg_nonhost_hoffA <- data.frame(do.call("rbind",reprops_veg_nonhost_hoffA))View(prop_veg_nonhost_hoffA$P_hoffA)#and then I made dataframe of the estimated proportion of the 4 groupsreprops_hosts_hoffA <- rbind.fill(prop_fl_host_hoffA,prop_fl_nonhost_hoffA,                                  prop_veg_host_hoffA,prop_veg_nonhost_hoffA)#and then I collected the data to multiply the counts and the proportionslabels <- c("fl_host","fl_nonhost","veg_host","veg_nonhost")type_hoffA <- rep(labels,each=200)bc_hoffA <- as.numeric(bc_hoffA$wpd)bb_hoffA <- as.numeric(reprops_hosts_hoffA$P_hoffA)#multiplying bootstrapped counts and bootstrapped proportionsest_hoffA <- bb_hoffA*bc_hoffA#make a dataframe againdf_est_hoffA <- data.frame(cbind(type_hoffA,                                 bc_hoffA,                                 bb_hoffA,                                 est_hoffA))#plot the  estimates just to checkdf_est_hoffA$est_hoffA <- as.numeric(as.character(df_est_hoffA$est_hoffA))#note that some values may not be numeric causing problems#plot_hoffA<-  ggplot(df_est_hoffA, aes(type_hoffA,est_hoffA))+  geom_boxplot()+  theme_classic()+  scale_y_continuous(trans = ss)+  labs(x=NULL,       y="estimated number of Pegoscapus hoffmeyeri A per 24h\n       (square-root transformed)")+  scale_x_discrete(limits = c('fl_host','fl_nonhost',                              'veg_host','veg_nonhost'),                   labels = c('receptive\nassociated\nUrostigma fig',                              'receptive\nother\nUrostigma fig',                              'vegetative\nassociated\nUrostigma fig',                              'vegetative\nother\nUrostigma fig'))#trying to sort the estimates per group#first the sorting didn’t work out well, but I found this ‘trick’ below#https://stackoverflow.com/questions/29399518/order-function-in-r-is-not-ordering-correctly/29401459df_est_hoffA$est_hoffA <- as.numeric(as.character(df_est_hoffA$est_hoffA))############# getting confidence intervals #########################flowering hoffA hosts#subset estimates for flowering hoffA hostsfl_host_est_hoffA <- subset(df_est_hoffA,type_hoffA=="fl_host")fl_host_est_hoffA<-  fl_host_est_hoffA[order(fl_host_est_hoffA$est_hoffA),]View(fl_host_est_hoffA)#calculating confidence intervals for flowering hostsfl_host_est_hoffA$est_hoffA[6] #0.255fl_host_est_hoffA$est_hoffA[195] #10.5####flowering hoffA non-hosts#subset estimates for flowering hoffA non-hostsfl_nonhost_est_hoffA <- subset(df_est_hoffA,type_hoffA=="fl_nonhost")fl_nonhost_est_hoffA<-  fl_nonhost_est_hoffA[order(fl_nonhost_est_hoffA$est_hoffA),]#calculating confidence intervals for flowering non-hostsfl_nonhost_est_hoffA$est_hoffA[6] #0fl_nonhost_est_hoffA$est_hoffA[195] #1.65####vegetative hoffA hosts#subset estimates for vegetative hoffA hostsveg_host_est_hoffA <- subset(df_est_hoffA,type_hoffA=="veg_host")veg_host_est_hoffA<-  veg_host_est_hoffA[order(veg_host_est_hoffA$est_hoffA),]#calculating confidence intervals for vegetative hostsveg_host_est_hoffA$est_hoffA[6] #0veg_host_est_hoffA$est_hoffA[195] #1.353333####vegetative hoffA non-hosts#subset estimates for vegetative hoffA non-hostsveg_nonhost_est_hoffA <- subset(df_est_hoffA,type_hoffA=="veg_nonhost")veg_nonhost_est_hoffA<-  veg_nonhost_est_hoffA[order(veg_nonhost_est_hoffA$est_hoffA),]#calculating confidence intervals for flowering non-hostsveg_nonhost_est_hoffA$est_hoffA[6] #0veg_nonhost_est_hoffA$est_hoffA[195] #0.03#################################################### estimate for Pegoscapus hoffmeyeri B ###########################################################  bootstrap count data ######AAFKE: I used this script that I found online and I modified it a bit# answer 6 from https://stackoverflow.com/questions/23831711/sample-n-random-rows-per-group-in-a-dataframe# This solution splits the data.frame into groups (fl_host, fl_nonhost, veg_host, AND veg_nonhost)# Then it samples 200 rows from each group with replacement.# Then I merged the list of data.frames into one dataframe.datac_hoffB <- lapply(split(datac, datac$for_hoffB),                      function(subdf) subdf[sample(1:nrow(subdf), 200, replace = TRUE),])bc_hoffB<-rbind(datac_hoffB$fl_host,datac_hoffB$fl_nonhost,                datac_hoffB$veg_host,datac_hoffB$veg_nonhost)#bc means “bootstrap count”data.frame(bc_hoffB)View(bc_hoffB)#and then I plotted these resampled counts (800 in total, 200 per group) to checkas.factor(bc_hoffB$for_hoffB)ggplot(bc_hoffB, aes(y=wpd,fill=for_hoffB))+  geom_boxplot()##### bootstrap proportion data ######to resample the barcoding data to get estimates of the proportions # I used the script of TomVanDooren but than on the groups (levels of factor) I added# I do the same steps 4 times for each group# (fl_host, fl_nonhost, veg_host, AND veg_nonhost)### 1 proportions for flowering hosts of hoffBsubdatprop_fl_host_hoffB<-subset(databar,for_hoffB=="fl_host")propresamples_fl_host_hoffB<-list()for(i in 1:200)propresamples_fl_host_hoffB[[i]]<-sample(factor(subdatprop_fl_host_hoffB$pollID),200,replace=T)# convert into proportions for hoffB hostsproper<-function(x){tab<-table(x);tab/sum(tab)}reprops_fl_host_hoffB<-lapply(propresamples_fl_host_hoffB,proper)View(reprops_fl_host_hoffB)#a made a data.frame with probablilities for hoffB hosts#since I don’t really know how to used these lists of dataframesprop_fl_host_hoffB <- data.frame(do.call("rbind",reprops_fl_host_hoffB))### 2 proportions for flowering non-hosts of hoffBsubdatprop_fl_nonhost_hoffB <-subset(databar,for_hoffB=="fl_nonhost")propresamples_fl_nonhost_hoffB<-list()for(i in 1:200)propresamples_fl_nonhost_hoffB[[i]]<-sample(factor(subdatprop_fl_nonhost_hoffB$pollID),200,replace=T)# convert into proportions for hoffB non-hostsproper<-function(x){tab<-table(x);tab/sum(tab)}reprops_fl_nonhost_hoffB<-lapply(propresamples_fl_nonhost_hoffB,proper)View(reprops_fl_nonhost_hoffB)#getting a data.frame with probablilities hoffB non-hostsprop_fl_nonhost_hoffB <- data.frame(do.call("rbind",reprops_fl_nonhost_hoffB))View(prop_fl_nonhost_hoffB$P_hoffB)### 3 proportions for vegetative hosts of hoffBsubdatprop_veg_host_hoffB<-subset(databar,for_hoffB=="veg_host")propresamples_veg_host_hoffB<-list()for(i in 1:200)propresamples_veg_host_hoffB[[i]]<-sample(factor(subdatprop_veg_host_hoffB$pollID),200,replace=T)# convert into proportions for hoffB non-hostsproper<-function(x){tab<-table(x);tab/sum(tab)}reprops_veg_host_hoffB<-lapply(propresamples_veg_host_hoffB,proper)View(reprops_veg_host_hoffB)#getting a data.frame with probablilities hoffB non-hostsprop_veg_host_hoffB <- data.frame(do.call("rbind",reprops_veg_host_hoffB))View(prop_veg_host_hoffB$P_hoffB)### 4 proportions for vegetative non-hosts of hoffBsubdatprop_veg_nonhost_hoffB<-subset(databar,for_hoffB=="veg_nonhost")propresamples_veg_nonhost_hoffB<-list()for(i in 1:200)propresamples_veg_nonhost_hoffB[[i]]<-sample(factor(subdatprop_veg_nonhost_hoffB$pollID),200,replace=T)# convert into proportions for hoffB non-hostsproper<-function(x){tab<-table(x);tab/sum(tab)}reprops_veg_nonhost_hoffB<-lapply(propresamples_veg_nonhost_hoffB,proper)View(reprops_veg_nonhost_hoffB)#getting a data.frame with probablilities hoffB non-hostsprop_veg_nonhost_hoffB <- data.frame(do.call("rbind",reprops_veg_nonhost_hoffB))View(prop_veg_nonhost_hoffB$P_hoffB)#and then I made dataframe of the estimated proportion of the 4 groupsreprops_hosts_hoffB <- rbind.fill(prop_fl_host_hoffB,prop_fl_nonhost_hoffB,                                  prop_veg_host_hoffB,prop_veg_nonhost_hoffB)#and then I collected the data to multiply the counts and the proportionslabels <- c("fl_host","fl_nonhost","veg_host","veg_nonhost")type_hoffB <- rep(labels,each=200)bc_hoffB <- as.numeric(bc_hoffB$wpd)bb_hoffB <- as.numeric(reprops_hosts_hoffB$P_hoffB)#multiplying bootstrapped counts and bootstrapped proportionsest_hoffB <- bb_hoffB*bc_hoffB#make a dataframe againdf_est_hoffB <- data.frame(cbind(type_hoffB,                                 bc_hoffB,                                 bb_hoffB,                                 est_hoffB))#plot the counts just to check#note that some values may not be numeric causing problemsdf_est_hoffB$est_hoffB <- as.numeric(as.character(df_est_hoffB$est_hoffB))#plot_hoffB<-  ggplot(df_est_hoffB, aes(type_hoffB,est_hoffB))+  geom_boxplot()+  theme_classic()+  scale_y_continuous(trans = ss)+  labs(x=NULL,       y="estimated number of Pegoscapus hoffmeyeri B per 24h\n       (square-root transformed)")+  scale_x_discrete(limits = c('fl_host','fl_nonhost',                              'veg_host','veg_nonhost'),                   labels = c('receptive\nassociated\nUrostigma fig',                              'receptive\nother\nUrostigma fig',                              'vegetative\nassociated\nUrostigma fig',                              'vegetative\nother\nUrostigma fig'))#trying to sort the estimates per group#first the sorting didn’t work out well, but I found this ‘trick’ below#https://stackoverflow.com/questions/29399518/order-function-in-r-is-not-ordering-correctly/29401459df_est_hoffB$est_hoffB <- as.numeric(as.character(df_est_hoffB$est_hoffB))############# getting confidence intervals #########################flowering hoffB hosts#subset estimates for flowering hoffB hostsfl_host_est_hoffB <- subset(df_est_hoffB,type_hoffB=="fl_host")fl_host_est_hoffB<-  fl_host_est_hoffB[order(fl_host_est_hoffB$est_hoffB),]View(fl_host_est_hoffB)#calculating confidence intervals for flowering hostsfl_host_est_hoffB$est_hoffB[6] #0.665fl_host_est_hoffB$est_hoffB[195] #24.15####flowering hoffB non-hosts#subset estimates for flowering hoffB non-hostsfl_nonhost_est_hoffB <- subset(df_est_hoffB,type_hoffB=="fl_nonhost")fl_nonhost_est_hoffB<-  fl_nonhost_est_hoffB[order(fl_nonhost_est_hoffB$est_hoffB),]#calculating confidence intervals for flowering non-hostsfl_nonhost_est_hoffB$est_hoffB[6] #0fl_nonhost_est_hoffB$est_hoffB[195] #3.3####vegetative hoffB hosts#subset estimates for vegetative hoffB hostsveg_host_est_hoffB <- subset(df_est_hoffB,type_hoffB=="veg_host")veg_host_est_hoffB<-  veg_host_est_hoffB[order(veg_host_est_hoffB$est_hoffB),]#calculating confidence intervals for vegetative hostsveg_host_est_hoffB$est_hoffB[6] #0veg_host_est_hoffB$est_hoffB[195] #2.9025####vegetative hoffB non-hosts#subset estimates for vegetative hoffB non-hostsveg_nonhost_est_hoffB <- subset(df_est_hoffB,type_hoffB=="veg_nonhost")veg_nonhost_est_hoffB<-  veg_nonhost_est_hoffB[order(veg_nonhost_est_hoffB$est_hoffB),]#calculating confidence intervals for vegetative non-hostsveg_nonhost_est_hoffB$est_hoffB[6] #0veg_nonhost_est_hoffB$est_hoffB[195] #0.165#################################################### estimate for Pegoscapus tonduzi ###########################################################  bootstrap counts ######AAFKE: I used this script that I found online and I modified it a bit# answer 6 from https://stackoverflow.com/questions/23831711/sample-n-random-rows-per-group-in-a-dataframe# This solution splits the data.frame into groups (fl_host, fl_nonhost, veg_host, AND veg_nonhost)# Then it samples 200 rows from each group with replacement.# Then I merged the list of data.frames into one dataframe.datac_tond <- lapply(split(datac, datac$for_tond),                     function(subdf) subdf[sample(1:nrow(subdf), 200, replace = TRUE),])bc_tond<-rbind(datac_tond$fl_host,datac_tond$fl_nonhost,               datac_tond$veg_host,datac_tond$veg_nonhost)#bc means “bootstrap count”data.frame(bc_tond)View(bc_tond)#and then I plotted these resampled counts (800 in total, 200 per group)as.factor(bc_tond$for_tond)ggplot(bc_tond, aes(y=wpd,fill=for_tond))+  geom_boxplot()##### bootstrap proportions ######## 1 proportions for flowering hosts of tondsubdatprop_fl_host_tond<-subset(databar,for_tond=="fl_host")propresamples_fl_host_tond<-list()for(i in 1:200)propresamples_fl_host_tond[[i]]<-sample(factor(subdatprop_fl_host_tond$pollID),200,replace=T)# convert into proportions for tond hostsproper<-function(x){tab<-table(x);tab/sum(tab)}reprops_fl_host_tond<-lapply(propresamples_fl_host_tond,proper)View(reprops_fl_host_tond)#a made a data.frame with probablilities for tond hosts#since I don’t really know how to used these lists of dataframesprop_fl_host_tond <- data.frame(do.call("rbind",reprops_fl_host_tond))### 2 proportions for flowering non-hosts of tondsubdatprop_fl_nonhost_tond <-subset(databar,for_tond=="fl_nonhost")propresamples_fl_nonhost_tond<-list()for(i in 1:200)propresamples_fl_nonhost_tond[[i]]<-sample(factor(subdatprop_fl_nonhost_tond$pollID),200,replace=T)# convert into proportions for tond non-hostsproper<-function(x){tab<-table(x);tab/sum(tab)}reprops_fl_nonhost_tond<-lapply(propresamples_fl_nonhost_tond,proper)View(reprops_fl_nonhost_tond)#getting a data.frame with probablilities tond non-hostsprop_fl_nonhost_tond <- data.frame(do.call("rbind",reprops_fl_nonhost_tond))View(prop_fl_nonhost_tond$P_tond)####################### 3 proportions for vegetative hosts of tondsubdatprop_veg_host_tond<-subset(databar,for_tond=="veg_host")propresamples_veg_host_tond<-list()for(i in 1:200)propresamples_veg_host_tond[[i]]<-sample(factor(subdatprop_veg_host_tond$pollID),200,replace=T)# convert into proportions for tond non-hostsproper<-function(x){tab<-table(x);tab/sum(tab)}reprops_veg_host_tond<-lapply(propresamples_veg_host_tond,proper)View(reprops_veg_host_tond)#getting a data.frame with probablilities tond non-hostsprop_veg_host_tond <- data.frame(do.call("rbind",reprops_veg_host_tond))View(prop_veg_host_tond$P_tond)####################### 4 proportions for vegetative non-hosts of tondsubdatprop_veg_nonhost_tond<-subset(databar,for_tond=="veg_nonhost")propresamples_veg_nonhost_tond<-list()for(i in 1:200)propresamples_veg_nonhost_tond[[i]]<-sample(factor(subdatprop_veg_nonhost_tond$pollID),200,replace=T)# convert into proportions for tond non-hostsproper<-function(x){tab<-table(x);tab/sum(tab)}reprops_veg_nonhost_tond<-lapply(propresamples_veg_nonhost_tond,proper)View(reprops_veg_nonhost_tond)#getting a data.frame with probablilities tond non-hostsprop_veg_nonhost_tond <- data.frame(do.call("rbind",reprops_veg_nonhost_tond))View(prop_veg_nonhost_tond$P_tond)#and then I made dataframe of the estimated proportion of the 4 groupsreprops_hosts_tond <- rbind.fill(prop_fl_host_tond,prop_fl_nonhost_tond,                                 prop_veg_host_tond,prop_veg_nonhost_tond)#and then I collected the data to multiply the counts and the proportionslabels <- c("fl_host","fl_nonhost","veg_host","veg_nonhost")type_tond <- rep(labels,each=200)bc_tond <- as.numeric(bc_tond$wpd)bb_tond <- as.numeric(reprops_hosts_tond$P_tond)#multiplying bootstrapped counts and bootstrapped proportionsest_tond <- bb_tond*bc_tond#make a dataframe againdf_est_tond <- data.frame(cbind(type_tond,                                bc_tond,                                bb_tond,                                est_tond))#plot the counts just to check#note that some values may not be numeric causing problemsdf_est_tond$est_tond <- as.numeric(as.character(df_est_tond$est_tond))#plot_tond <-ggplot(df_est_tond, aes(type_tond,est_tond))+  geom_boxplot()+  theme_classic()+  scale_y_continuous(trans = ss)+  labs(x=NULL,       y="estimated number of Pegoscapus tonduzii per 24h\n       (square-root transformed)")+  scale_x_discrete(limits = c('fl_host','fl_nonhost',                              'veg_host','veg_nonhost'),                   labels = c('receptive\nassociated\nUrostigma fig',                              'receptive\nother\nUrostigma fig',                              'vegetative\nassociated\nUrostigma fig',                              'vegetative\nother\nUrostigma fig'))#trying to sort the estimates per group#first the sorting didn’t work out well, but I found this ‘trick’ below#https://stackoverflow.com/questions/29399518/order-function-in-r-is-not-ordering-correctly/29401459df_est_tond$est_tond <- as.numeric(as.character(df_est_tond$est_tond))############# getting confidence intervals #########################flowering tond hosts#subset estimates for flowering tond hostsfl_host_est_tond <- subset(df_est_tond,type_tond=="fl_host")fl_host_est_tond<-  fl_host_est_tond[order(fl_host_est_tond$est_tond),]View(fl_host_est_tond)#calculating confidence intervals for flowering hostsfl_host_est_tond$est_tond[6] #19fl_host_est_tond$est_tond[195] #770####flowering tond non-hosts#subset estimates for flowering tond non-hostsfl_nonhost_est_tond <- subset(df_est_tond,type_tond=="fl_nonhost")fl_nonhost_est_tond<-  fl_nonhost_est_tond[order(fl_nonhost_est_tond$est_tond),]#calculating confidence intervals for flowering non-hostsfl_nonhost_est_tond$est_tond[6] #0fl_nonhost_est_tond$est_tond[195] #5.565####vegetative tond hosts#subset estimates for vegetative tond hostsveg_host_est_tond <- subset(df_est_tond,type_tond=="veg_host")veg_host_est_tond<-  veg_host_est_tond[order(veg_host_est_tond$est_tond),]#calculating confidence intervals for vegetative hostsveg_host_est_tond$est_tond[6] #0veg_host_est_tond$est_tond[195] #10.615####vegetative tond non-hosts#subset estimates for vegetative tond non-hostsveg_nonhost_est_tond <- subset(df_est_tond,type_tond=="veg_nonhost")veg_nonhost_est_tond<-  veg_nonhost_est_tond[order(veg_nonhost_est_tond$est_tond),]#calculating confidence intervals for vegetative non-hostsveg_nonhost_est_tond$est_tond[6] #0veg_nonhost_est_tond$est_tond[195] #0.06#add to each df_est a label for species namelab_gemA <- rep("P. gemellus A**", each = 800)df_est_gemA2<-cbind(lab_gemA,df_est_gemA)colnames(df_est_gemA2)<-c('species',                          'host_type',                          'bcount',                          'bbarc',                          'estimates')lab_gemB <- rep("P. gemellus B", each = 800)df_est_gemB2<-cbind(lab_gemB,df_est_gemB)colnames(df_est_gemB2)<-c('species',                          'host_type',                          'bcount',                          'bbarc',                          'estimates')lab_gemC <- rep("P. gemellus C", each = 800)df_est_gemC2<-cbind(lab_gemC,df_est_gemC)colnames(df_est_gemC2)<-c('species',                          'host_type',                          'bcount',                          'bbarc',                          'estimates')lab_hoffA <- rep("P. hoffmeyeri A", each = 800)df_est_hoffA2<-cbind(lab_hoffA,df_est_hoffA)colnames(df_est_hoffA2)<-c('species',                           'host_type',                           'bcount',                           'bbarc',                           'estimates')lab_hoffB <- rep("P. hoffmeyeri B", each = 800)df_est_hoffB2<-cbind(lab_hoffB,df_est_hoffB)colnames(df_est_hoffB2)<-c('species',                           'host_type',                           'bcount',                           'bbarc',                           'estimates')lab_tond <- rep("P. tonduzii", each = 800)df_est_tond2<-cbind(lab_tond,df_est_tond)colnames(df_est_tond2)<-c('species',                          'host_type',                          'bcount',                          'bbarc',                          'estimates')df_est_all<-rbind(df_est_gemA2,                  df_est_gemB2,                  df_est_gemC2,                  df_est_hoffA2,                  df_est_hoffB2,                  df_est_tond2)df_est_all$estimates <- as.numeric(as.character(df_est_all$estimates))View(df_est_all)#don't forget to create an Excel!!! [ESTIMATES_fromR_220707]####################################library("writexl")write_xlsx(df_est_all,"/Users/aafke/Dropbox/Mac/Desktop/estimates_220707.xlsx")#https://stackoverflow.com/questions/42588238/setting-individual-y-axis-limits-with-facet-wrap-not-with-scales-free-yggplot(data, aes(host_type,estimates))+  geom_boxplot()+  facet_wrap(~species,nrow = 3)+  scale_y_continuous(trans= scales::pseudo_log_trans(), breaks = c(1,10,100,1000),limits = c(0,8000))+  theme_bw()%+replace%  theme(panel.background = element_blank(),        panel.grid.major = element_blank(),         panel.grid.minor = element_blank(),        strip.background = element_blank())+  labs(x=NULL, y="estimated pollinator presence (N/24h)\nfor different Urostigma fig trees\n(pseudolog transformed)")+  scale_x_discrete(limits = c('fl_host','fl_nonhost','veg_host','veg_nonhost'),                   labels = c('species\nemerged\nfrom',                              'other\nspecies',                              'species\nemerged\nfrom',                              'other\nspecies'))+  theme(plot.margin = unit(c(.8,.8,.8,.8),"cm"))+  geom_signif(data=data.frame(species=c("P. gemellus B",                                        "P. hoffmeyeri A",                                        "P. hoffmeyeri B",                                        "P. tonduzii",                                        "P. tonduzii",                                        "P. tonduzii")),              aes(y_position=c(8.9,8.9,8.9,8.9,8,7),                  xmin=c(1,1,1,1,1,1),                  xmax=c(4,4,4,4,3,2),                  annotations=c(" ")),               manual = T)      #geom_signif(comparisons =list(c("fl_host", "fl_nonhost"),  #                              c("fl_host","veg_host"),   #                             c("fl_host","veg_nonhost")),    #          step_increase = 0.1,annotation = c("*"))
