# Supplementary material for: Field sampling of fig pollinator wasps across host species and host developmental phase: Implications for host recognition and specificity
Source: Ecol Evol. 2023 Sep 11;13(9):e10501. doi: 10.1002/ece3.10501 (PMC10495548; doi:10.1002/ece3.10501)
Supplement: Supplementary file 1 — Data S1. [file ECE3-13-e10501-s001.zip › SuppMaterial R script trapping data.rtf]

#AUTHORS Tom Van Dooren and Aafke Oldenbeuving#version January 13th 2023#aafke.oldenbeuving@naturalis.nl#import data and load packages#######################################library(pscl)library(glmmTMB)library(multcomp)# read in datadata<-read.csv(“pollinator_arrivals.csv")head(data)# date variable, numericaldata$datevals<-as.numeric(as.Date(data$date, "%d-%m-%y"))# zero-inflated mixed models on all observations################################################ different models fitted, with random effects on either lambda of the poisson or p of the binomial, or both or none.# offsets for the number of days a trap was open “Ndays”# ziformula gives fixed and random effects of the zero-inflation part# Nwasps: count, species: host species, date: date, type: host type.countsmmzif0<-glmmTMB(Nwasps~offset(log(Ndays))+type+(1|species)+(1|date),                      ziformula=~0,family=poisson,data=data) # no zero inflationcountsmmzif1<-glmmTMB(Nwasps~offset(log(Ndays))+type+(1|species)+(1|date),                      ziformula=~type,family=poisson,data=data) #zero inflation on typecountsmmzif2<-glmmTMB(Nwasps~offset(log(Ndays))+type+(1|species)+(1|date),                      ziformula=~type+(1|date),family=poisson,data=data) # zi on type and date (random)countsmmzif3<-glmmTMB(Nwasps~offset(log(Ndays))+type+(1|species)+(1|date),                      ziformula=~type+(1|species),family=poisson,data=data)  # zi on type and species (random)countsmmzif4<-glmmTMB(Nwasps~offset(log(Ndays))+type+(1|species),                      ziformula=~type,family=poisson,data=data) #zi on typecountsmmzif5<-glmmTMB(Nwasps~offset(log(Ndays))+type+(1|species),                      ziformula=~type+(1|date),family=poisson,data=data) # zi on type and date countsmmzif6<-glmmTMB(Nwasps~offset(log(Ndays))+type+(1|species),                      ziformula=~type+(1|species),family=poisson,data=data) #zi on type ant speciescountsmmzif7<-glmmTMB(Nwasps~offset(log(Ndays))+type+(1|date),                      ziformula=~type,family=poisson,data=data) #zi on type# no further models fitted: without random effects we would require the library below (glm with zero-inflated poisson distributions).# models 2,3 5, and 7 did not convergeAIC(countsmmzif0) #AIC 2867AIC(countsmmzif1) #AIC 2426AIC(countsmmzif2) #AIC NAAIC(countsmmzif3) #AIC NAAIC(countsmmzif4) #AIC 14630AIC(countsmmzif4) #AIC NA AIC(countsmmzif6) #AIC 14555AIC(countsmmzif7) #AIC NAsummary(countsmmzif1) # preferred model# fitted again without intercept, so all fixed effect parameters are group averagescountsmmzif1noint<-glmmTMB(Nwasps~offset(log(Ndays))+factor(type)+(1|species)+(1|date)-1,ziformula=~factor(type)-1,family=poisson,data=data)summary(countsmmzif1noint)# extra functions for glht()glht_glmmTMB <- function (model, ..., component="cond") {  glht(model, ...,       coef. = function(x) fixef(x)[[component]],       vcov. = function(x) vcov(x)[[component]],       df = NULL)}modelparm.glmmTMB <- function (model,                                coef. = function(x) fixef(x)[[component]],                               vcov. = function(x) vcov(x)[[component]],                               df = NULL, component="cond", ...) {  multcomp:::modelparm.default(model, coef. = coef., vcov. = vcov.,                               df = df, ...)}# overview and comparisons of parameters on modelconfint(countsmmzif1noint) # confidence intervals on model parameters, these are log transformed for counts and logit transformed for proportions zi.# repeat of output#                                     2.5 %     97.5 %  Estimate#cond.factor(type)non-fig         -6.0028891  0.0843859 -2.959252#cond.factor(type)receptive        0.9955020  2.4779614  1.736732#cond.factor(type)sterile         -2.0987472 -0.4418976 -1.270322#species.cond.Std.Dev.(Intercept)  0.7495665  1.8386004  1.173948#date.cond.Std.Dev.(Intercept)     1.4809525  1.9342398  1.692488#zi.factor(type)non-fig           -0.5218206  3.7258218  1.602001#zi.factor(type)receptive         -5.4586473 -2.4528711 -3.955759#zi.factor(type)sterile            0.4227186  1.0546255  0.738672confint(glht(countsmmzif1,linfct = mcp(type = "Tukey"))) # confidence intervals differences countsconfint(glht(countsmmzif1,linfct = mcp(type = "Tukey"),component="zi")) # confidence intervals differences proportions# zero-inflated mixed model and glm per species################################################ contrast matrices for pairwise comparisons on the zeroinfl() models belowKcounts <- rbind("Counts S - Rec" = c( 0, -1,  1,0),     # for models where only counts have a type effect (model 2)                 "Counts S - Rand" = c(0,  0,  1,0),                        "Counts Rec - Rand" = c( 0,  1, 0,0))Kcountsmodelwithprob <- rbind("Counts S - Rec" = c( 0, -1,  1,0,0,0),     # for models where counts and probabilities have a type effect (model 1, apply to conditional counts)                              "Counts S - Rand" = c(0,  0,  1,0,0,0),       	                              "Counts Rec - Rand" = c( 0,  1, 0,0,0,0))Kprobsmodelwithprob <- rbind("Probs S - Rec" = c( 0, 0,  0,0,-1,1),     # for models where counts and probabilities have a type effect (model 1, apply to zero-inflation part)                             "Probs S - Rand" = c(0,  0,  0,0,0,1),       	                             "Probs Rec - Rand" = c( 0,  0, 0,0,1,0))Kprobs <- rbind("Probs S - Rec" = c( 0,0, -1, 1),	# for models where only probabilities have a type effect (model 3, apply to zero-inflation part)                "Probs S - Rand" = c(0,0,0, 1),                "Probs Rec - Rand" = c( 0,0,1, 0))	# Ficus bullenei##########################datsubBULL<-subset(data,subset=species=="BULL"|species=="RAND")countsmmzifspBULL<-glmmTMB(Nwasps~offset(log(Ndays))+type+(1|species)+(1|date),                           ziformula=~type,family=poisson,data=datsubBULL) # no convergencecountsglmzifspBULL1<-zeroinfl(Nwasps~offset(log(Ndays))+type|type,data=datsubBULL)countsglmzifspBULL2<-zeroinfl(Nwasps~offset(log(Ndays))+type|1,data=datsubBULL)countsglmzifspBULL3<-zeroinfl(Nwasps~offset(log(Ndays))+1|type,data=datsubBULL)countsglmzifspBULL4<-zeroinfl(Nwasps~offset(log(Ndays))+1,data=datsubBULL)AIC(countsmmzifspBULL) #no convergenceAIC(countsglmzifspBULL1) # AIC 1388 <- lowestAIC(countsglmzifspBULL2) # AIC 1402AIC(countsglmzifspBULL3) # AIC 1769AIC(countsglmzifspBULL4) # AIC 1810confint(countsglmzifspBULL1) # confidence parameter estimates model#2.5 %      97.5 %#  count_(Intercept)   -3.6543286 -0.08255706#count_typereceptive  2.9396438  6.52164361#count_typesterile    1.1777845  4.76068663#zero_(Intercept)     0.4293256  4.01564909#zero_typereceptive  -5.3158031 -0.82372943#zero_typesterile    -1.9495396  1.73599788confint(glht(countsglmzifspBULL1,linfct=Kcountsmodelwithprob)) # estimates differences counts#Linear Hypotheses:#                         Estimate lwr     upr    #Counts S - Rec == 0    -1.7614  -1.9863 -1.5366#Counts S - Rand == 0    2.9692   0.9083  5.0302#Counts Rec - Rand == 0  4.7306   2.6702  6.7911confint(glht(countsglmzifspBULL1,linfct=Kprobsmodelwithprob)) #estimates contrasts probabilities#Linear Hypotheses:#                       Estimate lwr     upr    #Counts S - Rec == 0     2.9630   1.2845  4.6415#Counts S - Rand == 0   -0.1068  -2.2887  2.0752#Counts Rec - Rand == 0 -3.0698  -5.7292 -0.4103# Ficus colubrinae##########################datsubCOL<-subset(data,subset=species=="COL"|species=="RAND")countsmmzifspCOL<-glmmTMB(Nwasps~offset(log(Ndays))+type+(1|species)+(1|date),                          ziformula=~type,family=poisson,data=datsubCOL) # warning messagescountsglmzifspCOL1<-zeroinfl(Nwasps~offset(log(Ndays))+type|type,data=datsubCOL)countsglmzifspCOL2<-zeroinfl(Nwasps~offset(log(Ndays))+type|1,data=datsubCOL)countsglmzifspCOL3<-zeroinfl(Nwasps~offset(log(Ndays))+1|type,data=datsubCOL)countsglmzifspCOL4<-zeroinfl(Nwasps~offset(log(Ndays))+1,data=datsubCOL)AIC(countsmmzifspCOL) # AIC 98, but warning messages, singular convergencesummary(countsmmzifspCOL) #doesn't workconfint(countsmmzifspCOL) #doesn't workAIC(countsglmzifspCOL1) # AIC 159AIC(countsglmzifspCOL2) # AIC 158 <- lowest AICAIC(countsglmzifspCOL3) # AIC 373AIC(countsglmzifspCOL4) # AIC 398summary(countsglmzifspCOL2) #confint(countsglmzifspCOL2) ##                         2.5 %     97.5 %#  count_(Intercept)     -4.905438  -2.491158#count_typereceptive    5.429605   7.876810#count_typesterile   -212.636486 192.672973#zero_(Intercept)      -1.788816   1.181972confint(glht(countsglmzifspCOL2,linfct=Kcounts)) # because model 2 has lowest AIC#Linear Hypotheses:#                         Estimate  lwr       upr      #Counts S - Rec == 0     -16.6350 -248.0216  214.7517#Counts S - Rand == 0     -9.9818 -241.3700  221.4065#Counts Rec - Rand == 0    6.6532    5.2561    8.0503# Ficus costaricana##########################datsubCOS<-subset(data,subset=species=="COS"|species=="RAND")countsmmzifspCOS<-glmmTMB(Nwasps~offset(log(Ndays))+type+(1|species)+(1|date),                          ziformula=~type,family=poisson,data=datsubCOS) #convergence problemcountsglmzifspCOS1<-zeroinfl(Nwasps~offset(log(Ndays))+type|type,data=datsubCOS) countsglmzifspCOS2<-zeroinfl(Nwasps~offset(log(Ndays))+type|1,data=datsubCOS)countsglmzifspCOS3<-zeroinfl(Nwasps~offset(log(Ndays))+1|type,data=datsubCOS)countsglmzifspCOS4<-zeroinfl(Nwasps~offset(log(Ndays))+1,data=datsubCOS)AIC(countsmmzifspCOS) #NAAIC(countsglmzifspCOS1) #46.23AIC(countsglmzifspCOS2) #45.65AIC(countsglmzifspCOS3) #43.93 <- lowest AIC, only probabilitiesAIC(countsglmzifspCOS4) #44.36confint(countsglmzifspCOS3) # large confidence intervals?confint(glht(countsglmzifspCOS3,linfct=Kprobs))#Linear Hypotheses:#                         Estimate    lwr         upr        #Probs S - Rec == 0        27.6627 -14254.5537  14309.8792#Probs S - Rand == 0       14.6219 -14264.0150  14293.2589#Probs Rec - Rand == 0    -13.0408   -332.7876    306.7061# Ficus citrifolia##########################datsubCTR<-subset(data,subset=species=="CTR"|species=="RAND")countsmmzifspCTR<-glmmTMB(Nwasps~offset(log(Ndays))+(1|species)+type+(1|date),                          ziformula=~type,family=poisson,data=datsubCTR) #no convergencecountsglmzifspCTR1<-zeroinfl(Nwasps~offset(log(Ndays))+type|type,data=datsubCTR) #countsglmzifspCTR2<-zeroinfl(Nwasps~offset(log(Ndays))+type|1,data=datsubCTR)  #countsglmzifspCTR3<-zeroinfl(Nwasps~offset(log(Ndays))+1|type,data=datsubCTR)  #countsglmzifspCTR4<-zeroinfl(Nwasps~offset(log(Ndays))+1,data=datsubCTR)  #AIC(countsmmzifspCTR) # NAAIC(countsglmzifspCTR1) # AIC 3100.96 <- lowestAIC(countsglmzifspCTR2) # AIC 3116.126AIC(countsglmzifspCTR3) # AIC 13148.78AIC(countsglmzifspCTR4) # AIC 13177.93confint(glht(countsglmzifspCTR1,linfct=Kcountsmodelwithprob))# Linear Hypotheses:#                         Estimate lwr     upr    #Counts S - Rec == 0    -4.9478  -5.2238 -4.6717#Counts S - Rand == 0    2.4104   0.3283  4.4926#Counts Rec - Rand == 0  7.3582   5.2937  9.4227confint(glht(countsglmzifspCTR1,linfct=Kprobsmodelwithprob))#Linear Hypotheses:#                         Estimate   lwr        upr       #Probs S - Rec == 0       19.4798 -9203.8943  9242.8539#Probs S - Rand == 0      -2.0307    -4.2433     0.1818#Probs Rec - Rand == 0   -21.5105 -9244.8848  9201.8638# Ficus dugandii##########################datsubDUG<-subset(data,subset=species=="DUG"|species=="RAND")countsmmzifspDUG<-glmmTMB(Nwasps~offset(log(Ndays))+type+(1|species)+(1|date),                          ziformula=~type,family=poisson,data=datsubDUG) # no convergencecountsglmzifspDUG1<-zeroinfl(Nwasps~offset(log(Ndays))+type|type,data=datsubDUG) # countsglmzifspDUG2<-zeroinfl(Nwasps~offset(log(Ndays))+type|1,data=datsubDUG)  #countsglmzifspDUG3<-zeroinfl(Nwasps~offset(log(Ndays))+1|type,data=datsubDUG)  #countsglmzifspDUG4<-zeroinfl(Nwasps~offset(log(Ndays))+1,data=datsubDUG)  #AIC(countsmmzifspDUG) # NAAIC(countsglmzifspDUG1) # 568AIC(countsglmzifspDUG2) # 567 <- lowest AICAIC(countsglmzifspDUG3) # 1255AIC(countsglmzifspDUG4) # 1279confint(glht(countsglmzifspDUG2,linfct=Kcounts)) #Linear Hypotheses:#                         Estimate  lwr       upr      #Counts S - Rec == 0     -14.3678 -130.2180  101.4825#Counts S - Rand == 0     -6.3930 -122.2486  109.4627#Counts Rec - Rand == 0    7.9748    6.8516    9.0980# Ficus nymphaefolia##########################datsubNYM<-subset(data,subset=species=="NYM"|species=="RAND")countsmmzifspNYM<-glmmTMB(Nwasps~offset(log(Ndays))+type+(1|species)+(1|date),                          ziformula=~type,family=poisson,data=datsubNYM) #countsglmzifspNYM1<-zeroinfl(Nwasps~offset(log(Ndays))+type|type,data=datsubNYM) # countsglmzifspNYM2<-zeroinfl(Nwasps~offset(log(Ndays))+type|1,data=datsubNYM)  #countsglmzifspNYM3<-zeroinfl(Nwasps~offset(log(Ndays))+1|type,data=datsubNYM)  #countsglmzifspNYM4<-zeroinfl(Nwasps~offset(log(Ndays))+1,data=datsubNYM)  #AIC(countsmmzifspNYM) # 96 <- lowest AIC, but convergence problemAIC(countsglmzifspNYM1) # 103AIC(countsglmzifspNYM2) # 101 <- best model among glmAIC(countsglmzifspNYM3) # 116AIC(countsglmzifspNYM4) # 121summary(countsmmzifspNYM)confint(countsmmzifspNYM)confint(glht(countsglmzifspNYM2,linfct=Kcounts)) #Linear Hypotheses:#                         Estimate lwr     upr    #Counts S - Rec == 0    -3.4816  -5.5754 -1.3878#Counts S - Rand == 0    0.6947  -1.4553  2.8447#Counts Rec - Rand == 0  4.1763   2.5256  5.8269# Ficus obtusifolia##########################datsubOBT<-subset(data,subset=species=="OBT"|species=="RAND")countsmmzifspOBT<-glmmTMB(Nwasps~offset(log(Ndays))+type+(1|species)+(1|date),                          ziformula=~type,family=poisson,data=datsubOBT) #does not workcountsglmzifspOBT1<-zeroinfl(Nwasps~offset(log(Ndays))+type|type,data=datsubOBT) # countsglmzifspOBT2<-zeroinfl(Nwasps~offset(log(Ndays))+type|1,data=datsubOBT)  #countsglmzifspOBT3<-zeroinfl(Nwasps~offset(log(Ndays))+1|type,data=datsubOBT)  #countsglmzifspOBT4<-zeroinfl(Nwasps~offset(log(Ndays))+1,data=datsubOBT)  #AIC(countsmmzifspOBT) # NAAIC(countsglmzifspOBT1) # 362AIC(countsglmzifspOBT2) # 367AIC(countsglmzifspOBT3) # 736AIC(countsglmzifspOBT4) # 779confint(glht(countsglmzifspOBT1,linfct=Kcountsmodelwithprob)) #Linear Hypotheses:#                         Estimate lwr     upr    #Counts S - Rec == 0    -2.0676  -2.3452 -1.7899#Counts S - Rand == 0    2.4011   0.3306  4.4715#Counts Rec - Rand == 0  4.4686   2.4046  6.5326confint(glht(countsglmzifspOBT1,linfct=Kprobsmodelwithprob)) # #Linear Hypotheses:#                        Estimate   lwr        upr       #Probs S - Rec == 0     1.832e+01 -1.039e+04  1.042e+04#Probs S - Rand == 0   -3.186e+00 -5.468e+00 -9.039e-01#Probs Rec - Rand == 0 -2.151e+01 -1.043e+04  1.038e+04# Ficus paraensis##########################datsubPAR<-subset(data,subset=species=="PAR"|species=="RAND")countsmmzifspPAR<-glmmTMB(Nwasps~offset(log(Ndays))+type+(1|species)+(1|date),                          ziformula=~type,family=poisson,data=datsubPAR) #no convergencecountsglmzifspPAR1<-zeroinfl(Nwasps~offset(log(Ndays))+type|type,data=datsubPAR) # countsglmzifspPAR2<-zeroinfl(Nwasps~offset(log(Ndays))+type|1,data=datsubPAR)  #countsglmzifspPAR3<-zeroinfl(Nwasps~offset(log(Ndays))+1|type,data=datsubPAR)  #countsglmzifspPAR4<-zeroinfl(Nwasps~offset(log(Ndays))+1,data=datsubPAR)  #AIC(countsmmzifspPAR) # NAAIC(countsglmzifspPAR1) # 58.79AIC(countsglmzifspPAR2) # 58.22 <- lowest AICAIC(countsglmzifspPAR3) # 159.22AIC(countsglmzifspPAR4) # 169.03confint(glht(countsglmzifspPAR2,linfct=Kcounts)) # Linear Hypotheses:#                           Estimate  lwr       upr      #Counts S - Rec == 0     -15.8741 -860.1876  828.4393#Counts S - Rand == 0     -9.2030 -853.5171  835.1111#Counts Rec - Rand == 0    6.6711    5.5029    7.8393# Ficus perforata##########################datsubPERF<-subset(data,subset=species=="PERF"|species=="RAND")countsmmzifspPERF<-glmmTMB(Nwasps~offset(log(Ndays))+type+(1|species)+(1|date),                           ziformula=~type,family=poisson,data=datsubPERF) # does not workcountsglmzifspPERF1<-zeroinfl(Nwasps~offset(log(Ndays))+type|type,data=datsubPERF) # countsglmzifspPERF2<-zeroinfl(Nwasps~offset(log(Ndays))+type|1,data=datsubPERF)  # countsglmzifspPERF3<-zeroinfl(Nwasps~offset(log(Ndays))+1|type,data=datsubPERF)  # countsglmzifspPERF4<-zeroinfl(Nwasps~offset(log(Ndays))+1,data=datsubPERF)  # AIC(countsmmzifspPERF) # NAAIC(countsglmzifspPERF1) # 1173.312AIC(countsglmzifspPERF2) # 1172.566 <- lowest AICAIC(countsglmzifspPERF3) # 2273AIC(countsglmzifspPERF4) # 2319summary(countsglmzifspPERF2)confint(glht(countsglmzifspPERF2,linfct=Kcounts)) #Linear Hypotheses:#                           Estimate  lwr       upr      #Counts S - Rec == 0     -16.4994 -162.0231  129.0244#Counts S - Rand == 0     -8.1222 -153.6501  137.4057#Counts Rec - Rand == 0    8.3772    7.2293    9.5250# Ficus pertusa##########################datsubPERT<-subset(data,subset=species=="PERT"|species=="RAND")countsmmzifspPERT<-glmmTMB(Nwasps~offset(log(Ndays))+type+(1|species)+(1|date),                           ziformula=~type,family=poisson,data=datsubPERT) # does not workcountsglmzifspPERT1<-zeroinfl(Nwasps~offset(log(Ndays))+type|type,data=datsubPERT) # countsglmzifspPERT2<-zeroinfl(Nwasps~offset(log(Ndays))+type|1,data=datsubPERT)  # countsglmzifspPERT3<-zeroinfl(Nwasps~offset(log(Ndays))+1|type,data=datsubPERT)  # countsglmzifspPERT4<-zeroinfl(Nwasps~offset(log(Ndays))+1,data=datsubPERT)  # AIC(countsmmzifspPERT) # NAAIC(countsglmzifspPERT1) # 95AIC(countsglmzifspPERT2) # 94 <- lowest AICAIC(countsglmzifspPERT3) # 115AIC(countsglmzifspPERT4) # 126summary(countsglmzifspPERT2)confint(glht(countsglmzifspPERT2,linfct=Kcounts)) # Linear Hypotheses:#                           Estimate  lwr       upr      #Counts S - Rec == 0     -11.9357 -281.1926  257.3212#Counts S - Rand == 0     -6.9260 -276.1845  262.3324#Counts Rec - Rand == 0    5.0097    3.6879    6.3315# Ficus popenoei##########################datsubPOP<-subset(data,subset=species=="POP"|species=="RAND")countsmmzifspPOP<-glmmTMB(Nwasps~offset(log(Ndays))+(1|species)+type+(1|date),                          ziformula=~type,family=poisson,data=datsubPOP) # does not workcountsglmzifspPOP1<-zeroinfl(Nwasps~offset(log(Ndays))+type|type,data=datsubPOP) # countsglmzifspPOP2<-zeroinfl(Nwasps~offset(log(Ndays))+type|1,data=datsubPOP)  # countsglmzifspPOP3<-zeroinfl(Nwasps~offset(log(Ndays))+1|type,data=datsubPOP)  # countsglmzifspPOP4<-zeroinfl(Nwasps~offset(log(Ndays))+1,data=datsubPOP)  # AIC(countsmmzifspPOP) # 555, did not workAIC(countsglmzifspPOP1) # 6139AIC(countsglmzifspPOP2) # 6161AIC(countsglmzifspPOP3) # 13213AIC(countsglmzifspPOP4) # 13292confint(glht(countsglmzifspPOP1,linfct=Kcountsmodelwithprob)) ##Linear Hypotheses:#                         Estimate lwr     upr    #Counts S - Rec == 0    -4.1128  -4.2840 -3.9416#Counts S - Rand == 0    2.2835   0.2294  4.3377#Counts Rec - Rand == 0  6.3963   4.3482  8.4445confint(glht(countsglmzifspPOP1,linfct=Kprobsmodelwithprob)) ##Linear Hypotheses:#                           Estimate   lwr        upr       #Probs S - Rec == 0       19.2261 -7338.5317  7376.9838#Probs S - Rand == 0      -2.2844    -4.4186    -0.1503#Probs Rec - Rand == 0   -21.5105 -7379.2685  7336.2475# Ficus triangulo##########################datsubTRIA<-subset(data,subset=species=="TRIA"|species=="RAND")countsmmzifspTRIA<-glmmTMB(Nwasps~offset(log(Ndays))+type+(1|species)+(1|date),                           ziformula=~type,family=poisson,data=datsubTRIA) # does not workcountsglmzifspTRIA1<-zeroinfl(Nwasps~offset(log(Ndays))+type|type,data=datsubTRIA) # countsglmzifspTRIA2<-zeroinfl(Nwasps~offset(log(Ndays))+type|1,data=datsubTRIA)  # countsglmzifspTRIA3<-zeroinfl(Nwasps~offset(log(Ndays))+1|type,data=datsubTRIA)  # countsglmzifspTRIA4<-zeroinfl(Nwasps~offset(log(Ndays))+1,data=datsubTRIA)  # AIC(countsmmzifspTRIA) # NAAIC(countsglmzifspTRIA1) # 142.68AIC(countsglmzifspTRIA2) # 142.11 <- lowest AICAIC(countsglmzifspTRIA3) # 311AIC(countsglmzifspTRIA4) # 331confint(glht(countsglmzifspTRIA2,linfct=Kcounts)) # #Linear Hypotheses:#                           Estimate  lwr       upr      #Counts S - Rec == 0     -14.5233 -399.6227  370.5761#Counts S - Rand == 0     -7.8150 -392.9160  377.2859#Counts Rec - Rand == 0    6.7083    5.5734    7.8431# Ficus trigonata##########################datsubTRIG<-subset(data,subset=species=="TRIG"|species=="RAND")countsmmzifspTRIG<-glmmTMB(Nwasps~offset(log(Ndays))+type+(1|species)+(1|date),                           ziformula=~type,family=poisson,data=datsubTRIG) # convergence problemcountsglmzifspTRIG1<-zeroinfl(Nwasps~offset(log(Ndays))+type|type,data=datsubTRIG) # countsglmzifspTRIG2<-zeroinfl(Nwasps~offset(log(Ndays))+type|1,data=datsubTRIG)  # countsglmzifspTRIG3<-zeroinfl(Nwasps~offset(log(Ndays))+1|type,data=datsubTRIG)  # countsglmzifspTRIG4<-zeroinfl(Nwasps~offset(log(Ndays))+1,data=datsubTRIG)  # AIC(countsmmzifspTRIG) # NAAIC(countsglmzifspTRIG1) # 187.60AIC(countsglmzifspTRIG2) # 187.03 <- lowest AICAIC(countsglmzifspTRIG3) # 303AIC(countsglmzifspTRIG4) # 324summary(countsglmzifspTRIG2)confint(glht(countsglmzifspTRIG2,linfct=Kcounts)) # Linear Hypotheses:#                           Estimate  lwr       upr      #Counts S - Rec == 0     -15.8662 -264.2506  232.5182#Counts S - Rand == 0     -9.3048 -257.6916  239.0819#Counts Rec - Rand == 0    6.5614    5.4105    7.7122# Ficus turbinata##########################datsubTUR<-subset(data,subset=species=="TUR"|species=="RAND")countsmmzifspTUR<-glmmTMB(Nwasps~offset(log(Ndays))+type+(1|date),ziformula=~type,family=poisson,data=datsubTUR) # countsglmzifspTUR1<-zeroinfl(Nwasps~offset(log(Ndays))+type|type,data=datsubTUR) # countsglmzifspTUR2<-zeroinfl(Nwasps~offset(log(Ndays))+type|1,data=datsubTUR)  # countsglmzifspTUR3<-zeroinfl(Nwasps~offset(log(Ndays))+1|type,data=datsubTUR)  # countsglmzifspTUR4<-zeroinfl(Nwasps~offset(log(Ndays))+1,data=datsubTUR)  # AIC(countsmmzifspTUR) # 64AIC(countsglmzifspTUR1) # 62AIC(countsglmzifspTUR2) # 60 <- lowest AICAIC(countsglmzifspTUR3) # 136AIC(countsglmzifspTUR4) # 137summary(countsglmzifspTUR2)confint(countsglmzifspTUR2) # receptive > random, sterile > randomconfint(glht(countsglmzifspTUR2,linfct=Kcounts)) # receptive > random# Linear Hypotheses:#                       Estimate lwr      upr     # Counts S - Rec == 0    -1.16774 -2.30722 -0.02827# Counts S - Rand == 0    4.26073  2.22340  6.29806#Counts Rec - Rand == 0  5.42848  3.61499  7.24196
