# Supplementary material for: Field sampling of fig pollinator wasps across host species and host developmental phase: Implications for host recognition and specificity
Source: Ecol Evol. 2023 Sep 11;13(9):e10501. doi: 10.1002/ece3.10501 (PMC10495548; doi:10.1002/ece3.10501)
Supplement: Supplementary file 1 — Data S1. [file ECE3-13-e10501-s001.zip › SuppMaterial_phylos.docx]

**SUPPLEMENTAL MATERIAL**

Figure S1. Neighbor Joining Tree showing relationships among COI sequences from individual wasps collected from multiple fig species from the Neotropics. Outgroup sequences are *Tetrapus spp*., and ingroup sequences are *Pegoscapus spp*. Individuals collected from *Ficus bullenei* for this study are highlighted in red.

Figure S2. Neighbor Joining Tree showing relationships among COI sequences from individual wasps collected from multiple fig species from the Neotropics. Outgroup sequences are *Tetrapus spp*., and ingroup sequences are *Pegoscapus spp*. Individuals collected from *Ficus citrifolia* for this study are highlighted in red.

Figure S3. Neighbor Joining Tree showing relationships among COI sequences from individual wasps collected from multiple fig species from the Neotropics. Outgroup sequences are *Tetrapus spp*., and ingroup sequences are *Pegoscapus spp*. Individuals collected from *Ficus colubrinae* for this study are highlighted in red.

Figure S4. Neighbor Joining Tree showing relationships among COI sequences from individual wasps collected from multiple fig species from the Neotropics. Outgroup sequences are *Tetrapus spp*., and ingroup sequences are *Pegoscapus spp*. Individuals collected from *Ficus dugandiii* for this study are highlighted in red.

Figure S5. Neighbor Joining Tree showing relationships among COI sequences from individual wasps collected from multiple fig species from the Neotropics. Outgroup sequences are *Tetrapus spp*., and ingroup sequences are *Pegoscapus spp*. Individuals collected from *Ficus near trigonata* for this study are highlighted in red.

Figure S6. Neighbor Joining Tree showing relationships among COI sequences from individual wasps collected from multiple fig species from the Neotropics. Outgroup sequences are *Tetrapus spp*., and ingroup sequences are *Pegoscapus spp*. Individuals collected from *Ficus obtusifolia* for this study are highlighted in red.

Figure S7. Neighbor Joining Tree showing relationships among COI sequences from individual wasps collected from multiple fig species from the Neotropics. Outgroup sequences are *Tetrapus spp*., and ingroup sequences are *Pegoscapus spp*. Individuals collected from *Ficus paraensis* for this study are highlighted in red.

Figure S8. Neighbor Joining Tree showing relationships among COI sequences from individual wasps collected from multiple fig species from the Neotropics. Outgroup sequences are *Tetrapus spp*., and ingroup sequences are *Pegoscapus spp*. Individuals collected from *Ficus perforata* for this study are highlighted in red.

Figure S9. Neighbor Joining Tree showing relationships among COI sequences from individual wasps collected from multiple fig species from the Neotropics. Outgroup sequences are *Tetrapus spp*., and ingroup sequences are *Pegoscapus spp*. Individuals collected from *Ficus pertusa* for this study are highlighted in red.

Figure S10. Neighbor Joining Tree showing relationships among COI sequences from individual wasps collected from multiple fig species from the Neotropics. Outgroup sequences are *Tetrapus spp*., and ingroup sequences are *Pegoscapus spp*. Individuals collected from *Ficus popenoei* for this study are highlighted in red.

Figure S11. Neighbor Joining Tree showing relationships among COI sequences from individual wasps collected from multiple fig species from the Neotropics. Outgroup sequences are *Tetrapus spp*., and ingroup sequences are *Pegoscapus spp*. Individuals collected from *Ficus trigonata* for this study are highlighted in red.
